# Recovery and Characterization Studies of Post-Production Alloy Waste from the Automotive Industry

**DOI:** 10.3390/ma13245600

**Published:** 2020-12-08

**Authors:** Sylwester Żelazny, Witold Żukowski, Dariusz Bogdał, Szczepan Bednarz, Wiktor Kasprzyk, Tomasz Świergosz

**Affiliations:** 1 Department of Inorganic Chemistry, Faculty of Chemical Engineering and Technology, Cracow University of Technology, Warszawska 24, 31-155 Kraków, Poland; sylwester.zelazny@pk.edu.pl (S.Ż.); witold.zukowski@pk.edu.pl (W.Ż.); 2 Department of Biotechnology and Physical Chemistry, Faculty of Chemical Engineering and Technology, Cracow University of Technology, Warszawska 24, 31-155 Kraków, Poland; sbednarz@pk.edu.pl (S.B.); wiktor.kasprzyk@pk.edu.pl (W.K.); 3 Department of Analytical Chemistry, Faculty of Chemical Engineering and Technology, Cracow University of Technology, Warszawska 24, 31-155 Kraków, Poland

**Keywords:** alloy waste, Inconel 625, Inconel 718, Titanium Grade 5, thermal process, washing process, recovery, characterization, surface degradation, NMR, GC/MS, SEM-EDS

## Abstract

Superalloys provide high corrosion resistance and are widely used as high-performance materials in aerospace, automotive, chemical, and other industries. Herein, the investigation into the characteristics and properties of alloy waste; Inconel 625, Inconel 718, and Titanium Grade 5, from the automotive industry, was introduced as a result of a recovery in various processes. For this reason, the following procedures were carried as follows; the washing process to remove oil from the swarf was evaluated using several commercial agents and for the process of thermal disposal of processing fluids, a temperature of 900 °C was used in a muffle furnace without air access. The presented studies show that the commercially available series of washing agents did not modify the composition of the surface. However, the high temperatures during the calcination of oil residues are affecting the elemental composition of the alloys. According to the results of the analyses, it is not possible to remove 100% of the oil residues from alloy waste using washing agents based on light organic fractions; however, the effectiveness of this method reaches 99%. In this report, accurate SEM-EDS analyses show changes that occur on the surface after machining and removal of processing fluids. The NMR and GC/MS investigations indicate contaminants as a mixture of aliphatic and cycloaliphatic hydrocarbons with carbon numbers from C8–C30.

## 1. Introduction

The development of the automotive industry imposes the need to improve technologies and methods of producing highly demanding components [1,2,3]. The high-quality requirements placed on machine elements promote the design of resistant and durable materials, such as titanium and nickel alloys [4,5]. This type of materials maintains high pressures on processing. Currently, the most basic techniques of machining in the industry are cavity techniques such as turning, milling, drilling and grinding [6]. All the processes applied, produce significant amounts of swarf waste [7]. However, since no statistical information is available, the exact amount of swarf production worldwide remains unknown. The technical document states that only one of the three major car manufacturers has an annual production of over 40,000 tons of swarf [8]. Furthermore, it is claimed that using current techniques in metal processing, 10% of all the metals production is converted into swarf waste [9]. Moreover, waste alloy swarfs are estimated to be between 2.3 and 5.8 million tones based on the global use of metal removal fluids. Filtration and oil recycling systems are implemented to reduce the use of metal removal fluids and solid waste [10]. It is common procedure in the automobile industry to use different alloys with the high market value as the material for specific elements, such as Titanium Grade 5, Inconel 625, Inconel 718 [11]. The main waste stream is the swarf generated during the processing and coolants [8]. These swarfs need to be stored and recycled due to the extremely high costs of steel production. Swarf is currently classified as solid or hazardous waste, depending on its composition and legislative definitions in different countries. Therefore, they should be processed following the rules so that they can be reused as raw materials [12]. They should be thoroughly cleaned from fluids used in the treatment process. The type of machining (milling, turning, grinding) has a fundamental influence on the amount, form and type of contaminants generated during operating, mainly particulates [13,14,15]. No influence of machining type on values of the machining fluids parameters was found [16]. On the other hand, microbiological contamination of emulsion machining fluids is a problem concerning each stage of fluid exploitation [17]. No significant influence of machining type on the content of bacteria and fungi in liquids was determined [18]. The process of microbiological contamination is favored by the complex chemical composition of emulsion processing fluids [19]. Not without significance is determination of the pH value of working solutions, favorable for the processes of decomposition of liquid components by microorganisms [20]. Environmentally friendly technologies to reduce pollution and recover resources are being explored more recently to strengthen environmental legislation related to pollution [21]. Especially in the engineering and machining industry, interest in addressing recycling of metal swarf and cutting fluids [7,21].

Herein, the swarf waste from three alloys Inconel 625, Inconel 718 and Titanium Grade 5 as an inherent by-product of the automotive industry within the technique of metal recovery through washing and thermal processing of oil maximizes the value of the recovered alloys swarf and reduces the costs of the process. In this study, for the first time, waste from alloys after processing in the automobile industry was characterized. The waste was recovered in a washing treatment process using available solutions to become a reusable material. Data from the analysis of the material surface and elemental analysis were compared to the reference data of the originating materials to verify that the materials do not lose their initial value during the machining and treatment processes. The contamination generated by the cooling liquids during machining was also investigated and the most effective washing liquids were selected. Moreover, for the qualities of metals, a process of thermal oil disposal from the surface of the alloy’s swarf was carried out. Alloys have been characterized using available research techniques.

## 2. Materials and Methods

### 2.1. Recycling Materials

The swarfs from machining, i.e., alloys Inconel 625 (I625), Inconel 718 (I718), and Titanium Grade 5 (TG5), were received from the automobile company (Poznań, Great Poland, Poland). During the investigations, materials were used in the form received from the vehicle component manufacturer. In addition, the obtained materials had different sizes and forms including thickness and width (from less than 1.6 mm to more than 6.3 mm). Furthermore, the materials were covered with unknown oily substances derived from metalworking fluids.

### 2.2. Content of the Grain Fraction

To remove the organic parts from the alloys, samples of 100 g each were transferred into 2-L beakers, then poured 500 mL of acetone until complete immersion. The degreasing time was 3 h. After this time, the samples were successively seeped on a funnel with a quality filter Type 289. After the seepage, the samples together with the filter were placed in an oven and dried at 35 °C for 4 h. Afterwards, particle size distribution was carried out to illustrate the amount of different sized swarf generated in the machining process. The analysis was carried out on an apparatus LAB-06-300 produced by the Scientific and Technical Company.

### 2.3. Moisture Content

For determination of the swarfs physical properties, the analysis of moisture and oil content is the most crucial issue. For this purpose, a drying technique in a convection oven to constant moisture is required in accordance with existing specifications. According to the ASTM D2216-10 to determine moisture content, a portion of 20 g of each swarfs are placed in the oven and are performed at 110 ± 5 °C [22].

### 2.4. Oil Content

The method for the oil content of the material was performed in three repetitions. This was achieved by mixing 100 g of the sample dried in an oven with 500 mL of acetone and then shaking for 60 min. The liquid fraction was then decanted, and the precipitated swarfs were rinsed with 250 mL of water for 30 min by ultrasonic treatment. The above operations were repeated twice, and the solution was decanted. After the water treatment, the sample was dried in an oven at 110 °C for 3 h.

### 2.5. Scanning Electron Microscopy with Energy Dispersive X-Ray Spectroscopy (SEM–EDS)

The Scanning Electron Microscope (SEM) model TM3000 from Hitachi (Fukuoka, Japan) was used to observe the changes on the surface.

### 2.6. Gas Chromatography–Mass Spectrometry (GC-MS)

The gas chromatography (model: Agilent 7890B GC, Waltham, MA, USA) with mass spectrometry (model: Agilent 5977A GC/MSD) was used to verify the purity of the waste material in processes of washing and thermal treatment from oily substances before and after treatments to show what organic contaminants have to be dealt with. The GC/MS technique is overly sensitive and allows determining the degree of purification from organic compounds. Chromatographic analysis was performed to separate individual pollutants in extracted solutions allowing to define their mass spectra and the size of signals coming from individual compounds from the first and second stage of purification. The chromatographic separation program used the temperature increase of the chromatographic column (DB-5 Agilent) as follows: Initial temperature: 50 °C for 2 min and increase 10 °C/min to 250 °C. Inlet temperature 200 °C and injection volume 1 µL have been applied to all analyses in the investigation. The mass spectrometer worked in a full-scan mode in the *m/z* range from 10 to 600 u. Chromatograms were obtained from all the analyses. 

### 2.7. Nuclear Magnetic Resonance (^1^H NMR and ^13^C NMR)

The precision of NMR spectroscopy allows measuring the chemical shifts, thus obtaining information about chemical bonds and the structure of molecules occurring in oily liquid extracts. The analyses with the NMR technique were performed on a JEOL FT-NMR 500 MHz apparatus (JNM-ECZR500 RS1 version ECZR, Peabody, Massachusetts, USA

### 2.8. Oil Removal from Alloy Swarfs in Washing Process

Oil residues from alloy processing affect the possibility of metal recovery; therefore, a washing process was used to remove the oil from the swarfs. The choice of washing agents to remove contaminants from the swarfs was restricted according to the approval of the European market. The verification of the selected agents was determined regarding environmental safety. Oil removal from alloy swarf was carried out using the solvent-based solutions: Spirdane D25; Spirdane D40 and Spirdane D60. These organic solutions were selected after determining precisely which organic compounds must be treated. For washing process 100 g of the washing agent and 20 g of swarfs in a beaker was used. The complete mixture was performed with a mechanical stirrer with a speed of 120 rpm. After 20, 40, and 60 min, the swarf samples were taken, rinsed twice with distilled water, and dried in a laboratory dryer at 110 °C for 2 h. The purity of the material was analyzed on a scanning microscope with an EDS attachment.

### 2.9. Oil Removal from Alloy Swarfs in a Thermal Process

It is hazardous to utilize oil residues as part of hydrocarbon fuels on metal alloy swarfs since oil is explosive at high temperatures [23]. Nevertheless, the swarf samples of individual alloys were calcined at 900 °C in a muffle furnace to examine the level of contamination and structure of the alloy after high-temperature application treatment due to the low content of oil residue on the surface. The process of thermal removal each time involved 10 g of swarfs in an alumina ceramic crucible and lasted for 2 h at a given temperature. Afterwards, the swarfs were cooled down and analyzed using a scanning microscope with an EDS attachment.

## 3. Results and Discussion

**Figure 1:**
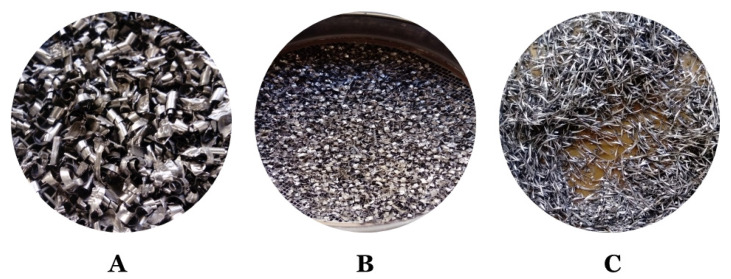
Illustration of a granulometric analysis using the dimensions of 6.3–3.15 mm Titanium Grade 5 (**A**), Inconel 625 (**B**), and Inconel 718 (**C**).

**Figure 2:**
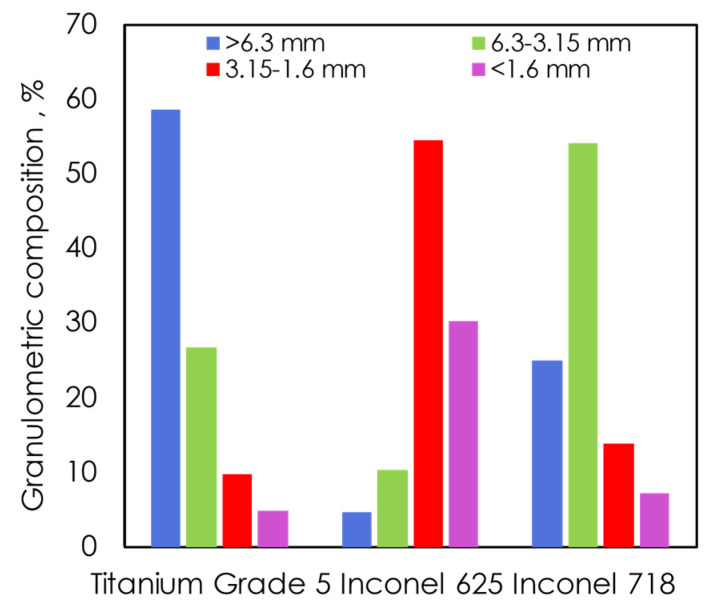
Granulometric composition of alloys after sieve analysis.

For all alloy waste, a clean surface is essential. The cleaning process must remove oils, greases, and traces of chemicals on the surface. Therefore, before the treatment, a number of analyses were performed to check parameters of waste and oily substances. Initially, the sieve analysis was performed on sieves with 1.6 mm; 3.15 mm; 6.3 mm diameters. Total sieving time was 5 min and 100 g of each of the swarfs were taken for analysis. The results of the analysis are presented in Table 1 and Figure 1. Since the swarfs constitute irregular shapes, a given grain fraction corresponds to the lowest diameter of a single swarf. Moreover, an exemplary graph of the granulometric analysis using the dimensions of 6.3–3.15 mm of TG5, I625, and I718 swarfs is shown in Figure 2.

Furthermore, based on the standard, the moisture content of the samples for TG5, I625 and I718 was 12 wt.%, 3.1 wt.%, and 3.4 wt.%, respectively, not changing in time after 2 and 3 h of drying. In addition, after one month of storage, the materials show the same moisture content as after receiving from automobile company (Table S1). Due to the type of method used to remove residues from the alloy surface, the amount of oil deposit in swarfs is important. Therefore, for this investigation, the oil content was determined by the gravimetric method during the solution-based washing removal. Following the findings of Ruffino and Zanetti [24]. As a result, the final mass of oil content was calculated through a comparison to the initial mass of each sample. The oil content of the swarf samples of TG5, I625, and I718 was 22 wt.%, 4.2 wt.%, and 4.6 wt.%, respectively. The respective values for moisture and oil content are shown in Table 2. In addition, after one month of storage, the material swarfs remained the same oil content (Table S1).

Based on the SEM photographs (Figure 2) at various magnifications, it was found that the research material comes from machining process. In this type of treatment, the swarfs are wrinkled as a result of the blade moving along the metal surface. Moreover, the distribution of machining fluid impurities are characterized by the dark stains on the SEM images.

**Figure 3:**
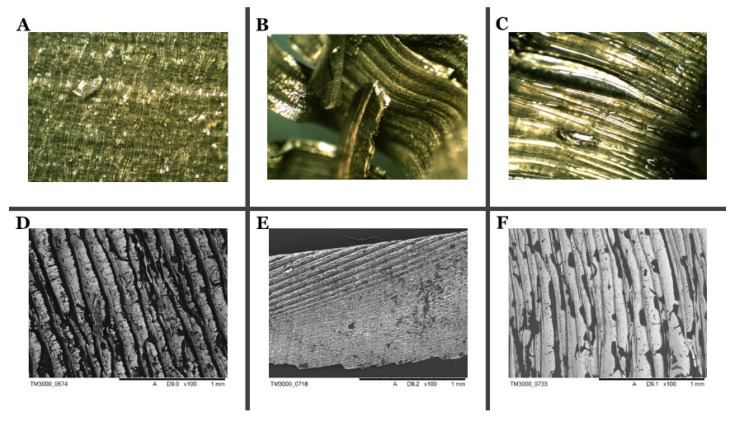
The surface of materials from SEM pictures: Titanium Grade 5; dark side, magnification 40× (**A**), Titanium Grade 5; dark side, magnification up to 1 mm (**D**), Inconel 625; dark side, magnification 40× (**B**), Inconel 625; dark side, magnification up to 1 mm (**E**), Inconel 718; dark side, magnification 40× (**C**), Inconel 625; dark side, magnification up to 1 mm (**F**).

The alloys surface distinguishes between the two sides due to the amount of oil accumulating on each side of the material (Figure S1). The bright side of the swarfs is characterized by a smaller surface wrinkle and impurities are much reduced and distributed relatively regularly over the entire metal surface. However, the dark side of the swarfs is wrinkled, and oil residues are mainly found in metal cavities and microcracks (Figure 3). Therefore, considering that, in the process of removing oily substances, the selection of proper cleaners plays a crucial role. Also, the EDS analysis confirms the existence of substantial changes between the two sides of the swarf surface. The EDS analysis of the dark area shows large coal accumulations. The analyzed area contains carbon and oxygen, which indicates the presence of large quantities of organic compounds in this area. The EDS analysis of the bright area shows that the carbon stock is much smaller; this indicates the presence of organic compounds in the area; however, organic contaminants cover the surface in smaller quantities. Due to the above, significant changes in the elemental analysis of a surface and oily impurities are noted. A detailed elemental analysis of the contaminants occur on the surface, as well as elemental analyses, are shown in Figures S2–S7.

For quantitative analysis of the chemical composition of alloy samples, the Optima 7300DV ICP-OES System from Perkin Elmer was used. Organic impurities were removed from the swarf by washing the samples in acetone several times. Analytical weights (1 g) were prepared from the cleaned and dried samples and solved in a mixture of nitric acid (60%) and hydrofluoric acid (40%) in a Perkin Elmer microwave mineralizer at 230 °C under the pressure of 35 atmospheres [25,26]. After dissolution, the sample was analyzed for the content of certain elements. The results of the analyses are presented in Table 3 below.

**Figure 4:**
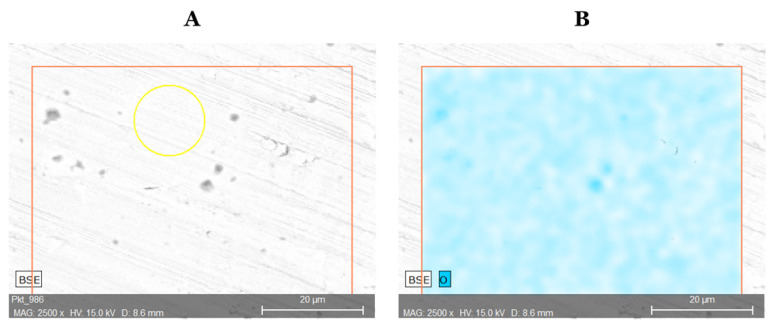
Analysis of the swarf surface of Inconel 718 (**A**); surface oxygen distribution of Inconel (**B**).

Identification of the surface composition by the SEM-EDS method was performed to compare to ICP-OES analysis results. The research material was prepared through several washings with acetone and then were placed in an oven and dried at 35 °C for 4 h. The elemental composition of the surface is shown in Table 4. To provide high results with the SEM-EDS method, a quantitative analysis (Figures S8–S10) was carried out on selected swarfs, which were smooth and their surface was polished [27]. In addition, in order to determine the extent to which the alloy has oxidized during machining, Figure 4 shows a surface analysis for distribution of oxygen. The oxygen content show high oxygen distribution on the swarf surface due to the oxidation of the alloy part during the machining. Following the values from the two selected methods (ICP-OES, SEM-EDS) of the elemental analysis they are considered as convergent.

For GC-MS analyses, the extraction of oily substances was carried out using 500 mL of solvent each time, in open beakers, since during the purification process, gases may lead to uncontrolled pressure escalation. However, during the experiments, no solvent evaporation was observed. Purification was intensified by immersion of beakers with solution and swarf material in an ultrasonic bath. Acetone 500 mL (AC) was selected for the extraction of oily substances from 100 g of the waste materials. The purification was carried out in two separate stages. The second stage of purification consisted of repeating the process with a new portion of the solvent. The second purification stage was introduced so that the purification efficiency of the first stage could be quantified by comparing the residual impurities in the solution after the second purification stage with the number of impurities (chromatographic peaks) in the solution from the first purification stage. The duration of each stage was 60 min. The extracted solutions were then analyzed for the content of extracted compounds. The peak areas obtained from the chromatographic analysis after the first purification are many times larger than the peak areas for the solutions after the second purification. Quantitative comparison of these surfaces allowed determining the degree of material purification. Metal surface treatment with solvents and ultrasounds from residues of oily substances allows removing organic compounds from the surfaces of the analyzed metals with efficiency from 98-99% (compared with the baseline of a pure solvent) (Figures S11–S13). The effectiveness of acetone removal is significant, as observed in the chromatograms of metal purification stages. The results obtained from GC/MS analyses of the oily extracts from I625, I718, and TG5 alloys show that they contain mainly a mixture of aliphatic and cycloaliphatic hydrocarbons with the number of carbon atoms ranging from C8–C30. The quality analyses were confirmed with 90% compatibility with the NIST database provided by the Agilent company. Additionally, for NMR analyses, an oily extract from the alloys surface has been extracted to prepare each time a sample of 40 mg/mL in CDCl_3_ + TMS. For each extract, ^13^C NMR and ^1^H NMR analyses were performed to determine functional groups and identify compounds present in working fluids. The spectra from the NMR analyses are shown in Figures S14–S19. The analysis of the NMR spectra shows that no significant chemical compounds are containing the following groups: Carboxylic, ketone, aldehyde (no signals > 165 ppm in the ^13^C NMR spectrum), aromatic derivatives (benzene, naphthalene type), and compounds containing double C=C bonds. The lack of <0.5 ppm signals also indicate the absence of organosilicon compounds: Poly (dimethylsiloxane) type. However, the spectra indicate the presence of aliphatic and cycloaliphatic hydrocarbons and possibly small amounts of hydroxyl groups -OH. The sample obtained from the extraction of I625 alloy contains aliphatic and cycloaliphatic hydrocarbons containing methylene, methine and methyl groups (0.5–1.9 ppm signals in the ^1^H NMR spectrum and 10–30 ppm in the ^13^C spectrum). The samples obtained from the extraction of I718 and TG5 alloys have a similar composition (Figure S20). Process of washing alloy waste from the oil residues, to be reusable for industry, with solvent-based solutions was carried out using Spirdane D25, Spirdane D40, Spirdane D60. These solvents are called a white spirit consisting of complex hydrocarbon substances obtained by hydro-refining a crude oil cut (n-alkanes, isoalkanes). These solvents offer toxicological and ecotoxicological levels below values specified by legislation in force. Moreover, the solvents are especially designed for the degreasing industry or applications requiring a fast evaporation rate. Additionally, the degree of washing is related to oil content (Table 2). The sieve analysis shows that the highest amount of oil on the surface contains TG5 and has the highest adhesion to the alloy surface (Table 1). The oil content is also directly related to the degree of washing resulting in the lowest degree for TG5 washing (Table 5). Following a series of investigations into the surface washing using the Spirdane cleaning solution, the contamination was practically completely removed from the surface of the alloy (Figure 4 and Figures S21–S29). The longer the time it takes to wash the swarfs in the solvents, the more effective the surface treatment is. However, there are only residual amounts of contamination on the bright (smoother) side, which is probably since the blade of the cutting machine more permanently left the contamination on the surface. In addition, no clusters of washing liquids used during cleaning have been observed on the surface (Figures S21–S29). The percentage of oil residues was determined using GC/MS technique according to the protocol used to identify the type of contaminations. Analyses have shown that washing with solvents for 60 min almost completely removes surface contamination. The removal range for oily substances is between 93.7 and 98.6 percent, indicating high process efficiency (Table 5). Solvent-based washing processes give the same results in three repetitions. Effective washing capacity depends primarily on its ability to disperse, transport, dissolve, and thus remove impurities from the solid matrix [10]. However, it is considered that the reason why the oil removal efficiency decreases with an increase in specific mass density is that the sample molecules are more agglomerated as the mass density increases, making contact difficult between the molecules and the washing agents. This means that the specific gravity has played a significant role in removing oil from the alloy swarfs [28].

**Figure 5:**
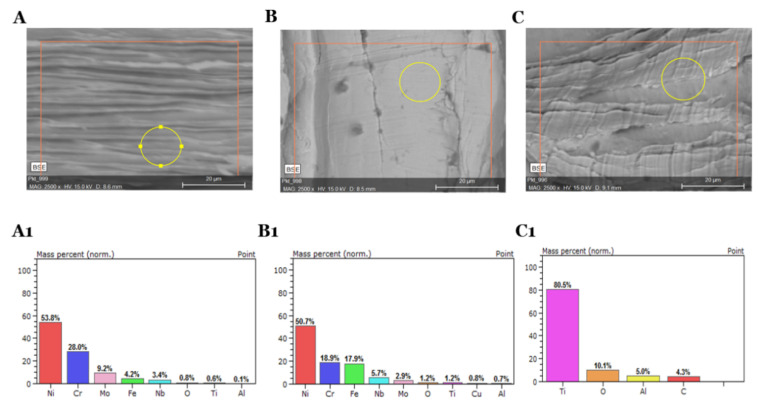
SEM-EDS photos after oil removal from Inconel 625 (**A**,**A1**), Inconel 718 (**B**,**B1**), Titanium Grade 5 (**C**,**C1**) swarfs in aqueous treatment process (Spirdane D40 after 60 min) with the elemental composition of the surfaces.

In addition, the microcracks that form during the alloying process create areas where the oil enters deeper, resulting in exceedingly difficult for the washing agent to penetrate and wash out contaminants. Even a long washing process does not eliminate 100% of the oil residues. It has been observed that washing the surfaces with solvents has a higher or lower oxygen distribution than in the case of untreated material. (Table 4 and Table 5). However, the remaining composition of the surface stays unchanged (Table 6 and Figure 5). This shows that the solvents used for washing do not cause oxidation of the alloys’ surfaces and can be employed as washing agent to recover alloys for re-use, while maintaining their original properties.

**Figure 6:**
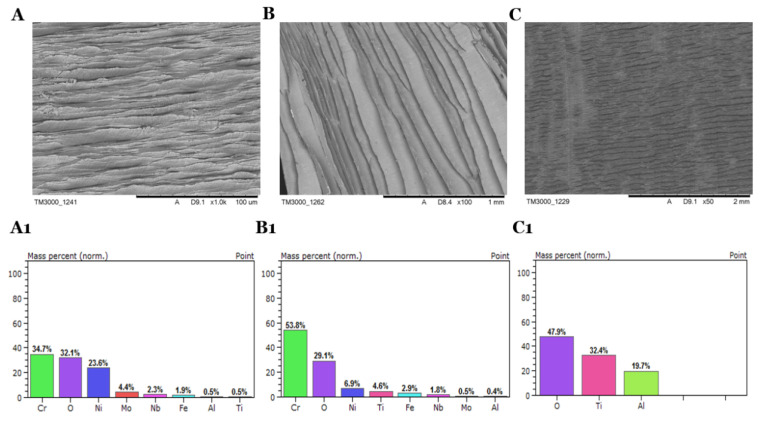
SEM-EDS photos after oil removal from Inconel 625 (**A**,**A1**), Inconel 718 (**B**,**B1**), Titanium Grade 5 (**C**,**C1**) swarfs in a thermal process (900 °C) with the elemental composition of the surfaces.

In the process of thermal removal of oil residues at temperature 900 °C in the muffle furnace, changes in the structure of the alloys and significant oxygen content on the surface were observed (Figure 6). Due to the lack of oxygen availability in the muffle furnace, the pyrolysis caused the oxidation on the surface. The main components of the alloys have been reduced in significant quantities. On the other hand, there is a high oxygen distribution on the surface, reaching almost 50% for TG5 (Table 6). In the process of thermal removal of oil residues, the microcracks formed on both sides of the materials have an impact on the high-temperature oil, whose main component is carbon, which reduces the composition on the surface of the alloys. The enormous changes in the composition of the alloys do not allow the use of a thermal treatment process to dispose of oil residues. The materials treated in this manner cannot be fully recovered for reapplication.

Although it is not possible to remove 100% of the oil residues employing washing with solvent-based detergents, it is advantageous to obtain a material with a degree of purification close to 100% for use to other applications. Mishra et al. for the recovery of ferrous grinding swarf used also commercial detergents achieving 100% of cleaning effectiveness [29]. However, the studies available in the literature are not based on effectiveness verification based on the GCMS technique and therefore cannot be compared with other results. In this case, a combination of heavy organic fractions is better to clean the metal surface with light organic fractions such as Spirdane D25. The results of the presented analyses show that it is possible to dispose of up to 99% of the contaminants in a short time. Therefore, the above studies, due to the surface coverage with oxygen in small amount after washing, make the process of passivation more convenient and it can be carried out to maintain the material’s corrosion resistance. A standard treatment process slightly reduces the corrosion resistance of the materials. The passivation process first removes free iron, which may be on the surface from contact with machine tools or oil residuals. Cleaning procedures of I625, I728, and TG5, reported in these studies. are sufficient to start the passivation process in alkaline solutions. The lack of available alloy cleaning techniques in the literature shows that this issue is worth investigation.

## 4. Conclusions

The feasibility of the washing process and thermal treatment for swarf recovery into reusable materials for the industry was investigated. Microscopic analysis of I625, I718, and TG5 have shown that the swarf surfaces differ in texture. Besides, the analysis showed that the impurities are irregularly distributed, and in the case of the wrinkled side (the dark side) the impurities are mainly located in its cavities. The contaminants contain mainly carbon indicating that the covering substances are mainly aliphatic and cycloaliphatic hydrocarbons, with no nitrogen. The gravimetric analysis showed that the content of impurities is about 4.2%, 4.6%, and 22% for I625, I718, and TG5, respectively, and the amount of impurities does not change during storage. Additionally, during the particle size analysis, discrepancies in the particle size were found, with the highest percentage fractions in the range 3.5–1.6 mm (I625), 6.3–3.15 mm (I718) and above 6.3 mm (TG5). Attempts to use washing agents show that organic solvents have a good impact on the surface structure and washes oil residues with almost 99% of effectiveness. Also, it has been found that the thermal treatment of I625, I718, and TG5 alloys at 900 °C removes contaminants; oxidizing significantly the surface of the alloys and leads to irreversible changes in structure.

## Figures and Tables

**Table 1:** Granulometric composition of alloy swarfs: Titanium Grade 5, Inconel 625, Inconel 718 in percentage after previous removal of organic parts from the surface.

Sieves (mm)	Content of the Grain Fraction [%]		
	Titanium Grade 5	Inconel 625	Inconel 718
>6.3	58.53	4.73	24.93
6.3–3.15	26.82	10.41	54.05
3.15–1.6	9.76	54.57	13.81
<1.6	4.89	30.29	7.21

**Table 2:** Respective values for physical analysis of swarf materials.

Alloy Type	Titanium Grade 5	Inconel 625	Inconel 718
Oil Content	22 wt.%	4.2 wt.%	4.6 wt.%
Moisture Content	12 wt.%	3.1 wt.%	3.4 wt.%

**Table 3:** The Inductively Coupled Plasma Optical Emission Spectrometry (ICP-OES) elemental results of alloy swarfs analyses.

Titanium Grade 5		Inconel 625		Inconel 718	
Elements	wt.%	Elements	wt.%	Elements	wt.%
Ti	82.79	Ni	59.21	Ni	53.92
Al	6.89	Cr	21.17	Cr	18.17
V	4.48	Mo	9.60	Fe	17.78
Sb	1.90	Fe	4.29	Mo	4.50
Co	0.23	Al	1.36	Nb	2.87
Fe	0.22	Sb	0.74	Ti	1.05
Cr	0.04	Pb	0.48	Al	0.52
Mn	0.04	Ti	0.38	Co	0.41
Cd	0.01	Zr	0.06	Sb	0.37
		Mn	0.04	Pb	0.27
		Cu	0.03	Zr	0.12
				Cu	0.05

**Table 4:** The SEM-EDS elemental results of alloy swarfs surface analyses.

Titanium Grade 5		Inconel 625		Inconel 718	
Elements	wt.%	Elements	wt.%	Elements	wt.%
Ti	80.40	Ni	53.80	Ni	54.50
O	12.20	Cr	28.00	Fe	19.50
Al	5.20	Mo	9.20	Cr	18.90
C	2.00	Fe	4.20	Mo	6.10
		Nb	3.40	Al	0.70
		O	0.80	O	0.20
		Ti	0.60		
		Al	0.10		

**Table 5:** Removal percentages of oil residues from swarfs after 60 min using washing agents.

Alloy Type	Titanium Grade 5	Inconel 625	Inconel 718
Washing agent	Oil removal [%]	Oil removal [%]	Oil removal [%]
SPIRDANE D25	96.2	98.6	97.8
SPIRDANE D40	94.6	96.4	96.7
SPIRDANE D60	93.7	95.8	95.9

**Table 6:** Oxygen distribution on the surface of swarfs in aqueous treatment process and thermal treatment process.

Alloy Type	Titanium Grade 5	Inconel 625	Inconel 718
Treatment Process Type	Oxygen [%]	Oxygen [%]	Oxygen [%]
Aqueous Treatment Process	10.1	0.8	1.2
Thermal Treatment Process	47.9	32.1	29.1

## Supplementary Materials

The following are available online at https://www.mdpi.com/1996-1944/13/24/5600/s1, Table S1. Relevant values for physical analysis of swarf materials after one month of storage. Figure S1. The surface of swarf materials from SEM pictures: Titanium Grade 5; dark side; magnification up to 500 µm (A), Titanium Grade 5; bright side; magnification up to 500 µm (D), Inconel 625; dark side; magnification up to 500 µm (B), Inconel 625 bright side; magnification up to 500 µm (E), Inconel 718; dark side; magnification up to 500 µm (C), Inconel 625; bright side; magnification up to 500 µm (F). Figure S2. SEM image of Inconel 718—the dark side—magnification up to 20 µm (A-B). EDS elemental analyses (C-D). Figure S3. SEM image of Inconel 718—the bright side—magnification up to 20 µm (A-B). EDS elemental analyses (C-D). Figure S4. SEM image of Inconel 625—the dark side—magnification up to 20 µm (A-B). EDS elemental analyses (C-D). Figure S5. SEM image of Inconel 625—the bright side—magnification up to 20 µm (A-B). EDS elemental analyses (C-D). Figure S6. SEM image of Titanium Grade 5—the dark side—magnification up to 10 µm (A-B). EDS elemental analyses (C-D). Figure S7. SEM image of Titanium Grade 5—the bright side—magnification up to 10 µm (A-B). EDS elemental analyses (C-D). Figure S8. SEM-EDS analysis on cleaned swarf surface of Titanium Grade 5 alloy at the selected point. Figure S9. SEM-EDS analysis on cleaned swarf surface of Inconel 625 alloy at the selected point. Figure S10. SEM-EDS analysis on cleaned swarf surface of Inconel 718 alloy at the selected point. Figure S11. Chromatogram of acetone oily extract by GC/MS technique for Inconel 625. First metal ultrasonic treatment. Figure S12. Chromatogram of acetone oily extract by GC/MS technique for Inconel 718. First metal ultrasonic treatment. Figure S13. Chromatogram of acetone oily extract by GC/MS technique for Inconel 625, 718 and Titanium Grade 5. Second metal ultrasonic treatment. Figure S14. The 13C NMR spectrum obtained by extraction of the organic fraction of the Inconel 625 alloy. Figure S15. The 1H NMR spectrum obtained by extraction of the organic fraction of the Inconel 625 alloy. Figure S16. The 13C NMR spectrum obtained by extraction of the organic fraction of the Inconel 718 alloy. Figure S17. The 1H NMR spectrum obtained by extraction of the organic fraction of the Inconel 718 alloy. Figure S18. The 13C NMR spectrum obtained by extraction of the organic fraction of the Titanium Grade 5 alloy. Figure S19. The 1H NMR spectrum obtained by extraction of the organic fraction of the Titanium Grade 5 alloy. Figure S20. The comparison of 1H NMR spectra of the analyzed organic fractions: (A) Inconel 625; (B) Inconel 718; (C) Titanium Grade 5, indicates their high chemical similarity. Figure S21. SEM-EDS photos after oil removal from Inconel 625 swarfs by washing with Spirdane D25 after 20 (A-dark side; A1-bright side), 40 (B-dark side; B1-bright side), and 60 (C-dark side; C1-bright side) minutes. Figure S22. SEM-EDS photos after oil removal from Inconel 718 swarfs by washing with Spirdane D25 after 20 (A-dark side; A1-bright side), 40 (B-dark side; B1-bright side), and 60 (C-dark side; C1-bright side) minutes. Figure S23. SEM-EDS photos after oil removal from Titanium Grade 5 swarfs by washing with Spirdane D25 after 20 (A-dark side; A1-bright side), 40 (B-dark side; B1-bright side), and 60 (C-dark side; C1-bright side) minutes. Figure S24. SEM-EDS photos after oil removal from Inconel 625 swarfs by washing with Spirdane D40 after 20 (A-dark side; A1-bright side), 40 (B-dark side; B1-bright side), and 60 (C-dark side; C1-bright side) minutes. Figure S25. SEM-EDS photos after oil removal from Inconel 718 swarfs by washing with Spirdane D40 after 20 (A-dark side; A1-bright side), 40 (B-dark side; B1-bright side), and 60 (C-dark side; C1-bright side) minutes. Figure S26. SEM-EDS photos after oil removal from Titanium Grade 5 swarfs by washing with Spirdane D40 after 20 (A-dark side; A1-bright side), 40 (B-dark side; B1-bright side), and 60 (C-dark side; C1-bright side) minutes. Figure S27. SEM-EDS photos after oil removal from Inconel 625 swarfs by washing with Spirdane D60 after 20 (A-dark side; A1-bright side), 40 (B-dark side; B1-bright side), and 60 (C-dark side; C1-bright side) minutes. Figure S28. SEM-EDS photos after oil removal from Inconel 718 swarfs by washing with Spirdane D60 after 20 (A-dark side; A1-bright side), 40 (B-dark side; B1-bright side), and 60 (C-dark side; C1-bright side) minutes. Figure S29. SEM-EDS photos after oil removal from Titanium Grade 5 swarfs by washing with Spirdane D60 after 20 (A-dark side; A1-bright side), 40 (B-dark side; B1-bright side), and 60 (C-dark side; C1-bright side) minutes.
