# Supplementary material for: Recovery and Characterization Studies of Post-Production Alloy Waste from the Automotive Industry
Source: Materials (Basel). 2020 Dec 8;13(24):5600. doi: 10.3390/ma13245600 (PMC7763364; doi:10.3390/ma13245600)
Supplement: Supplementary file 1 [file materials-13-05600-s001.pdf]

supplementary

Recovery and Characterization Studies of Post-Production Alloy Waste from the Automotive Industry

Sylwester Żelazny [1], Witold Żukowski [1], Dariusz Bogdał [2,*], Szczepan Bednarz [2], Wiktor Kasprzyk [2] and Tomasz Świergosz [3,*]

[1] Department of Inorganic Chemistry, Faculty of Chemical Engineering and Technology, Cracow University of Technology, Warszawska 24, 31-155 Kraków, Poland; sylwester.zelazny@pk.edu.pl (S.Z.); witold.zukowski@pk.edu.pl (W.Z.)
[2] Department of Biotechnology and Physical Chemistry, Faculty of Chemical Engineering and Technology, Cracow University of Technology, Warszawska 24, 31-155 Kraków, Poland; sbednarz@pk.edu.pl (S.B.); wiktor.kasprzyk@pk.edu.pl (W.K.)
[3] Department of Analytical Chemistry, Faculty of Chemical Engineering and Technology, Cracow University of Technology, Warszawska 24, 31-155 Kraków, Poland
* Correspondence: pcbogdal@cyf-kr.edu.pl (D.B.); tomasz.swiergosz@pk.edu.pl (T.Ś.)

Received: 12 November 2020; Accepted: 6 December 2020; Published: 8 December 2020

Table S1. Relevant values for physical analysis of swarf materials after one month of storage.

Alloy type	Titanium Grade 5	Inconel 625	Inconel 718
Oil Content	22 wt.%	4.2 wt.%	4.6 wt.%
Moisture Content	12 wt.%	3.1 wt.%	3.4 wt.%

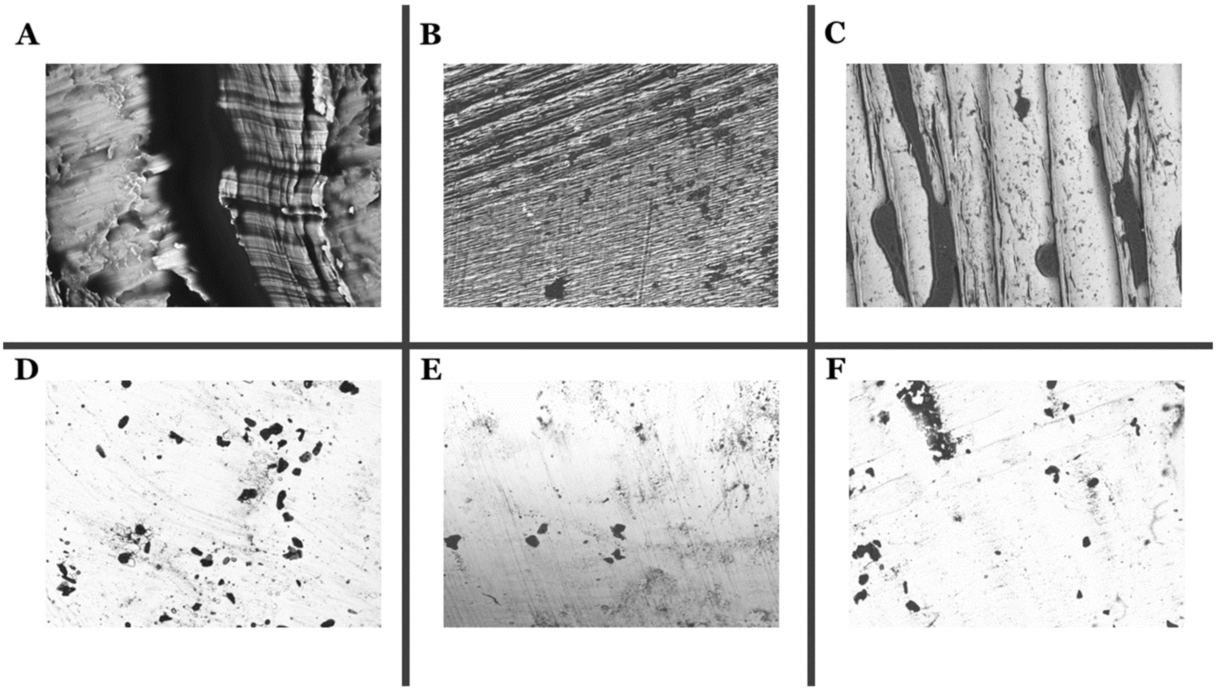

Figure S1. The surface of swarf materials from SEM pictures: Titanium Grade 5; dark side; magnification up to 500 μm (**A**), Titanium Grade 5; bright side; magnification up to 500 μm (**D**),

Inconel 625; dark side; magnification up to 500 µm (**B**), Inconel 625 bright side; magnification up to 500 µm (**E**), Inconel 718; dark side; magnification up to 500 µm (**C**), Inconel 625; bright side; magnification up to 500 µm (**F**).

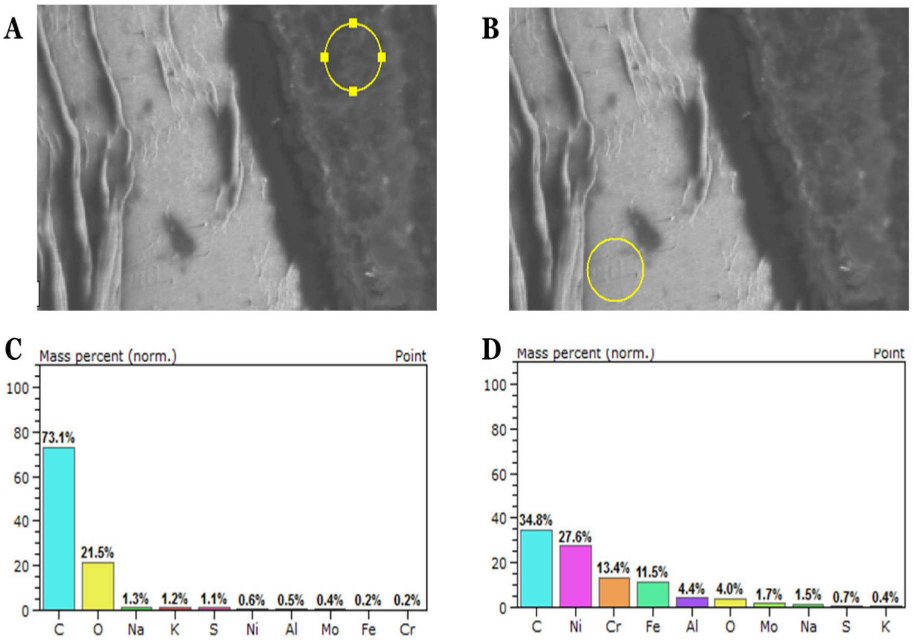

Figure S2. SEM image of Inconel 718 - the dark side - magnification up to 20 µm (**A**-**B**). EDS elemental analyses (**C**-**D**).

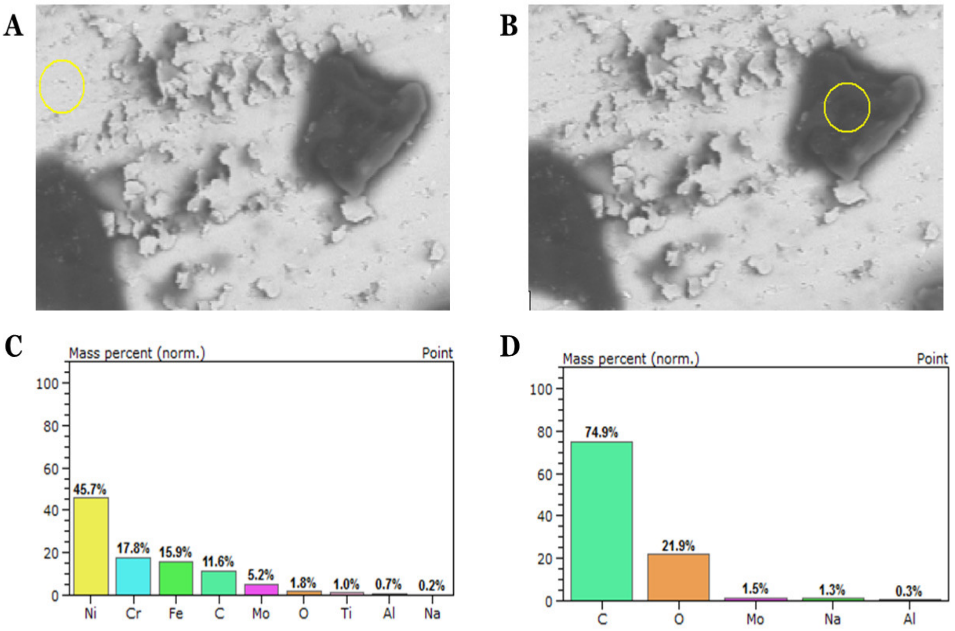

Figure S3. SEM image of Inconel 718 - the bright side - magnification up to 20 µm (**A**-**B**). EDS elemental analyses (**C**-**D**).

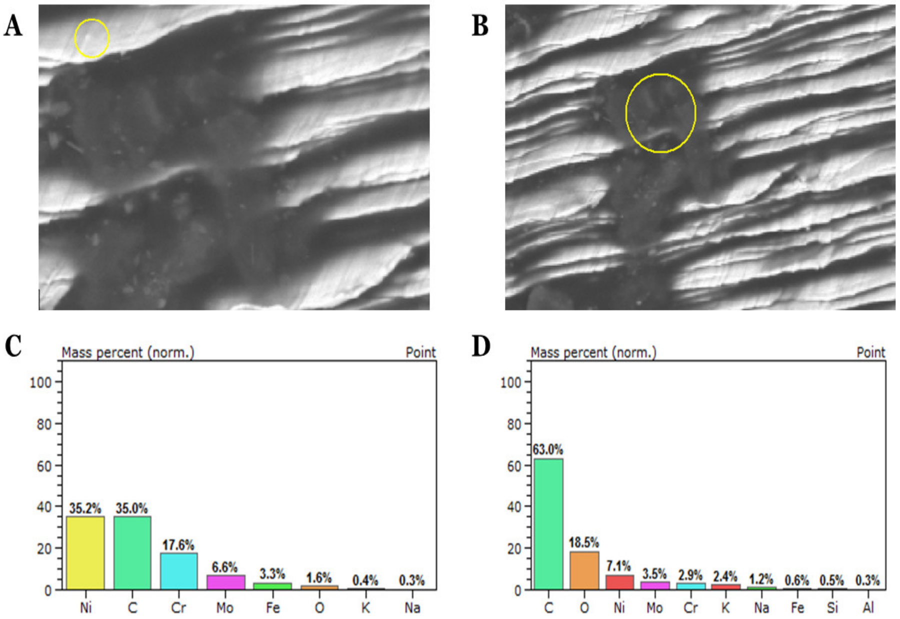

Figure S4. SEM image of Inconel 625 - the dark side - magnification up to 20 μm (**A**-**B**). EDS elemental analyses (**C**-**D**).

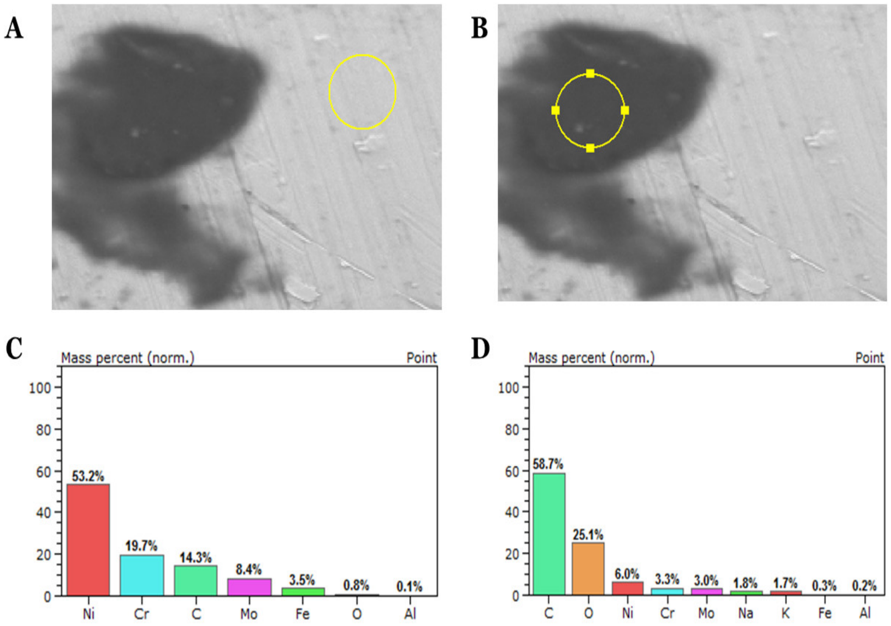

Figure S5. SEM image of Inconel 625 - the bright side - magnification up to 20 μm (**A**-**B**). EDS elemental analyses (**C**-**D**).

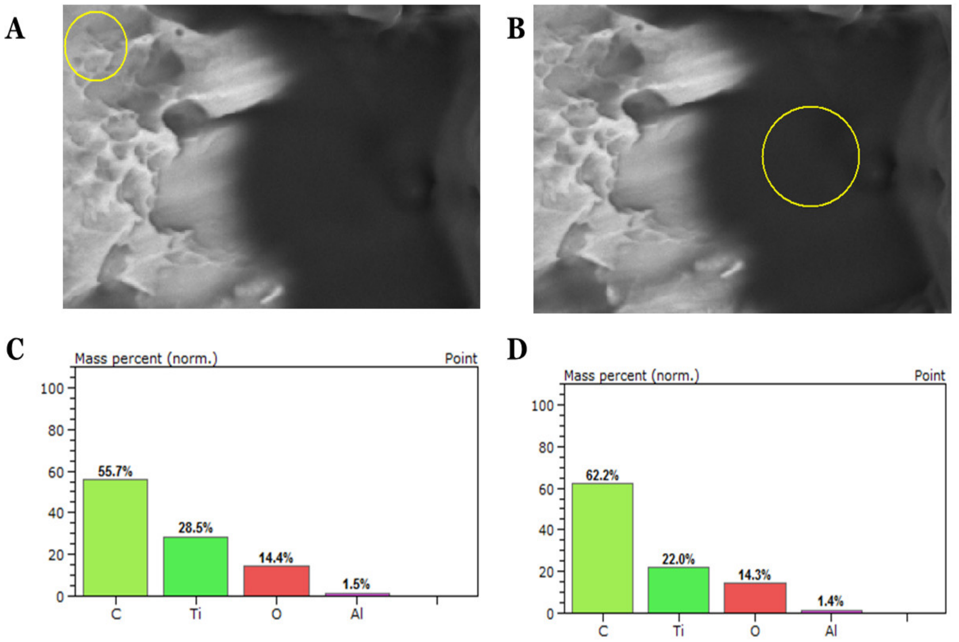

Figure S6. SEM image of Titanium Grade 5 - the dark side - magnification up to 10 µm (**A**-**B**). EDS elemental analyses (**C**-**D**).

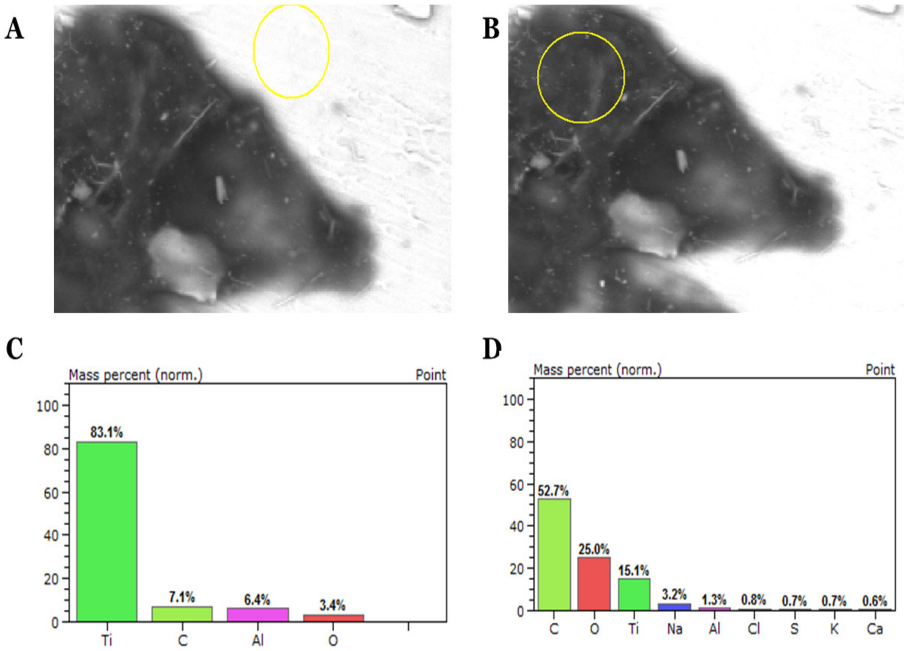

Figure S7. SEM image of Titanium Grade 5 - the bright side - magnification up to 10 µm (**A**-**B**). EDS elemental analyses (**C**-**D**).

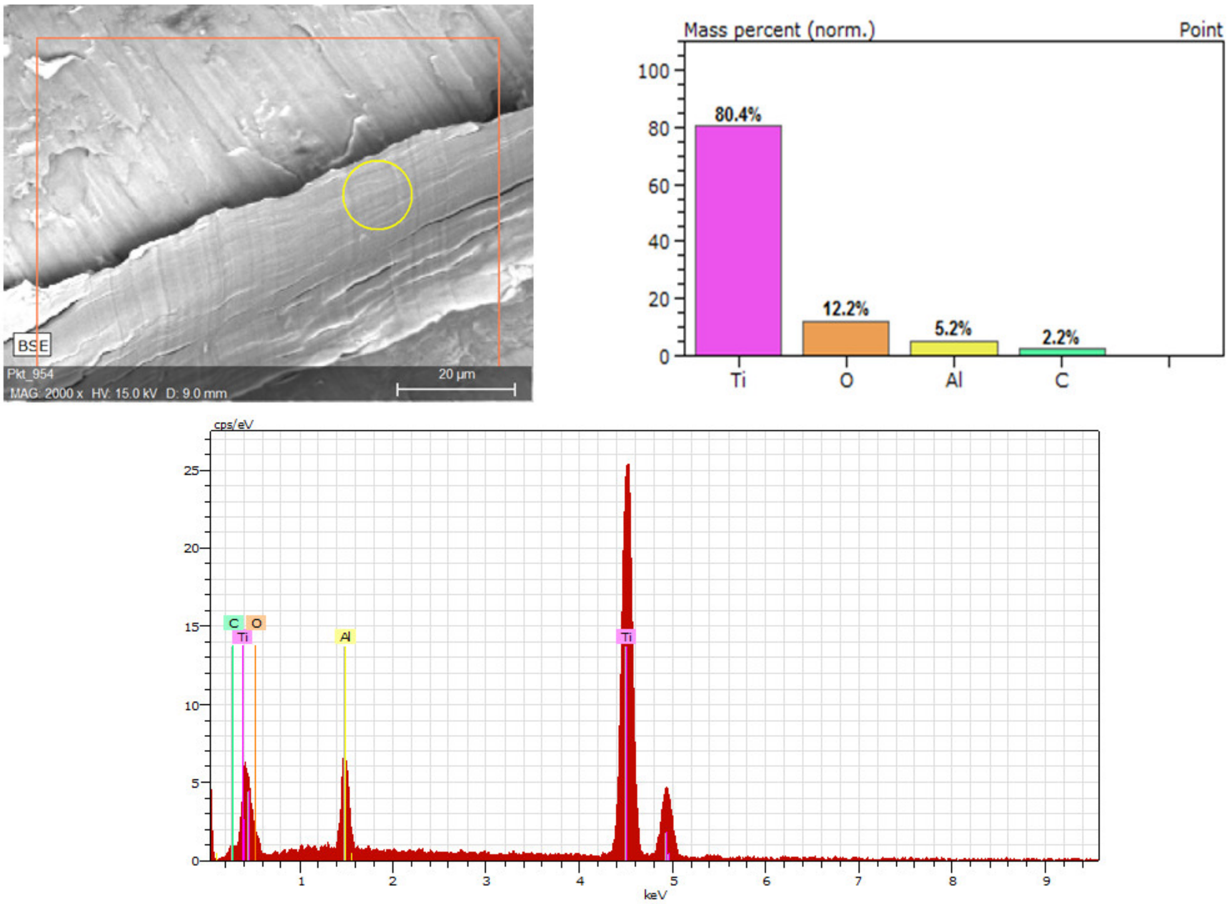

Figure S8. SEM-EDS analysis on cleaned swarf surface of Titanium Grade 5 alloy at the selected point.

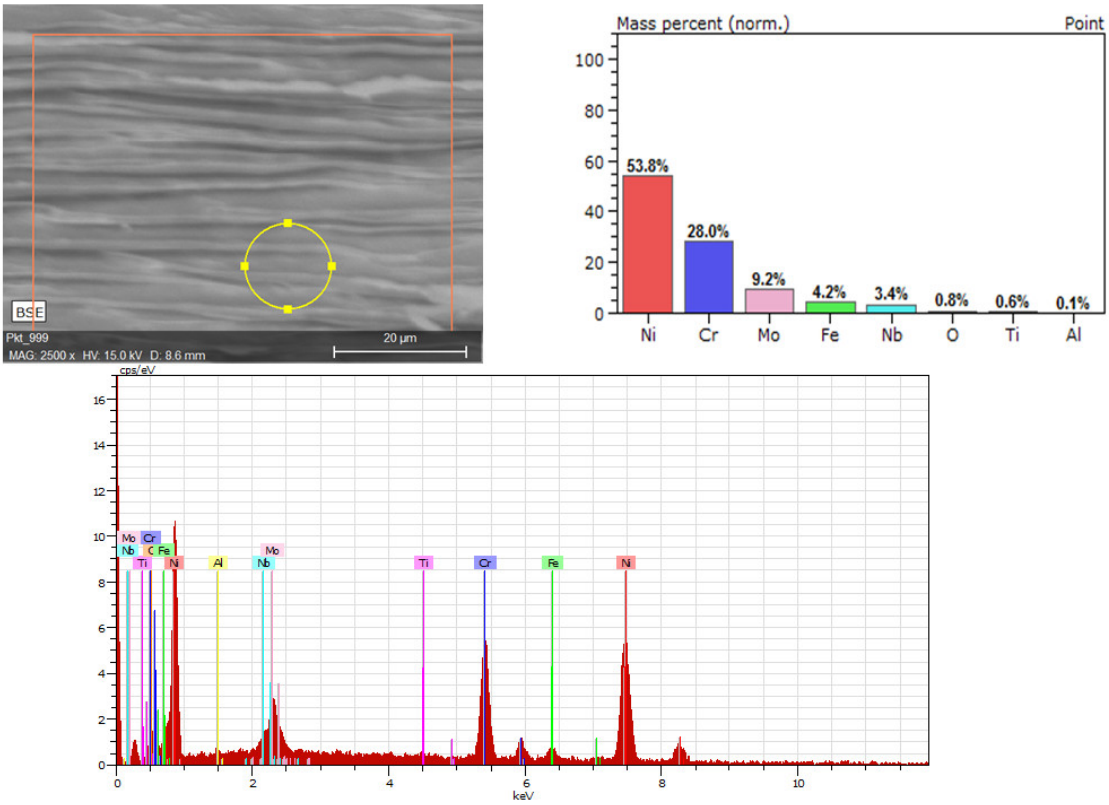

Figure S9. SEM-EDS analysis on cleaned swarf surface of Inconel 625 alloy at the selected point.

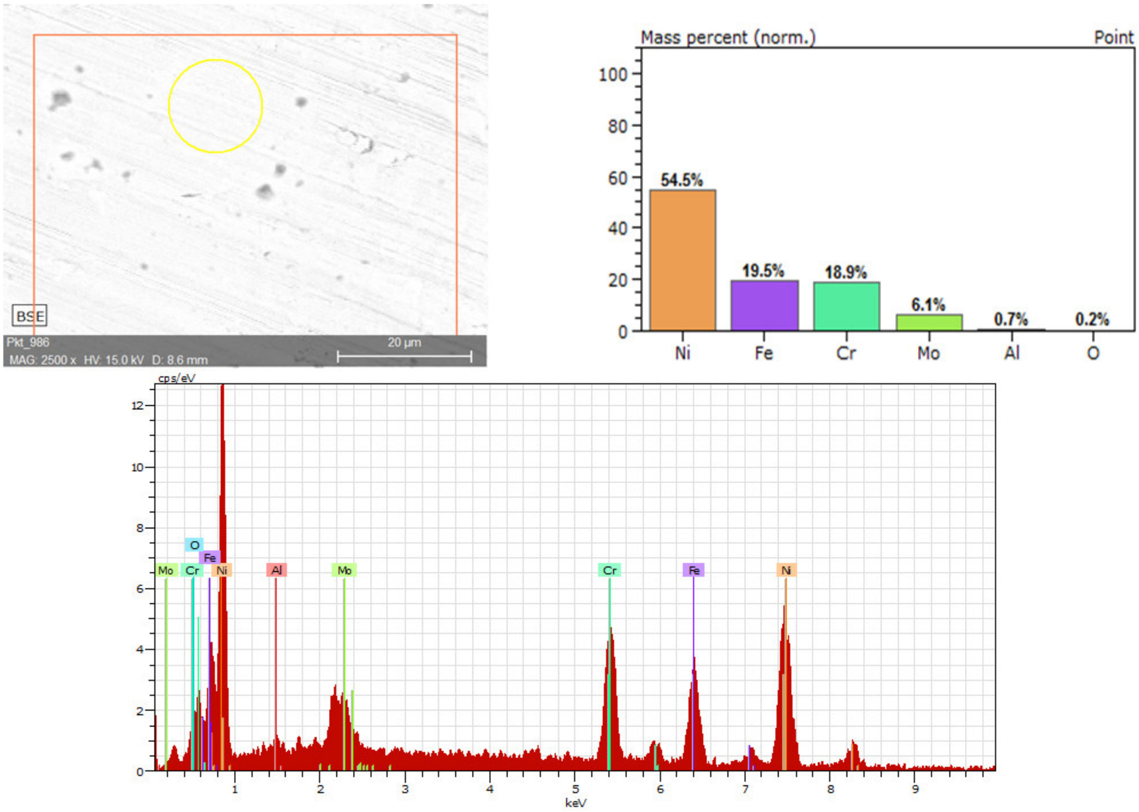

Figure S10. SEM-EDS analysis on cleaned swarf surface of Inconel 718 alloy at the selected point.

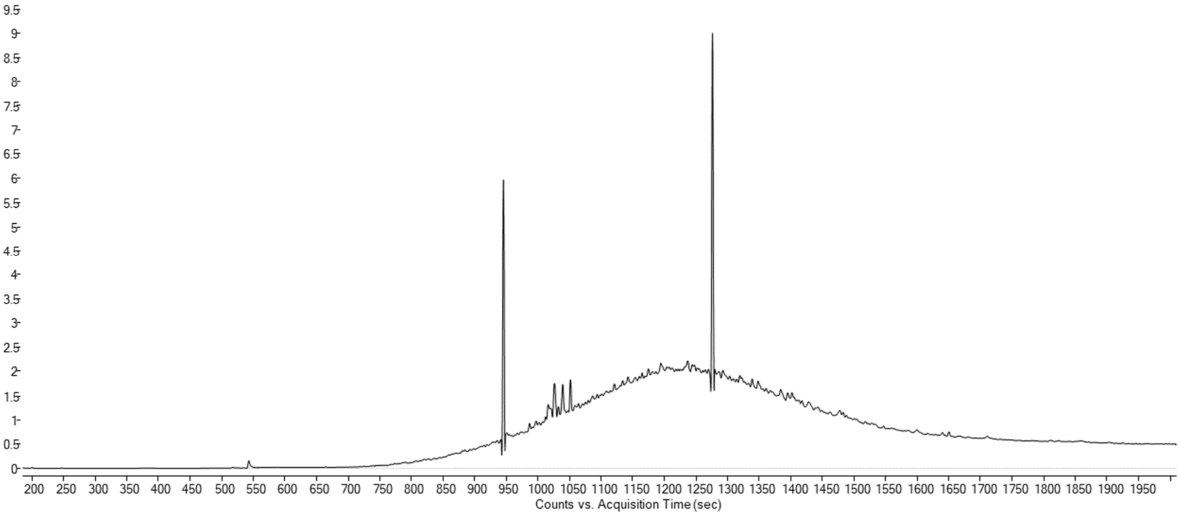

Figure S11. Chromatogram of acetone oily extract by GC/MS technique for Inconel 625. First metal ultrasonic treatment.

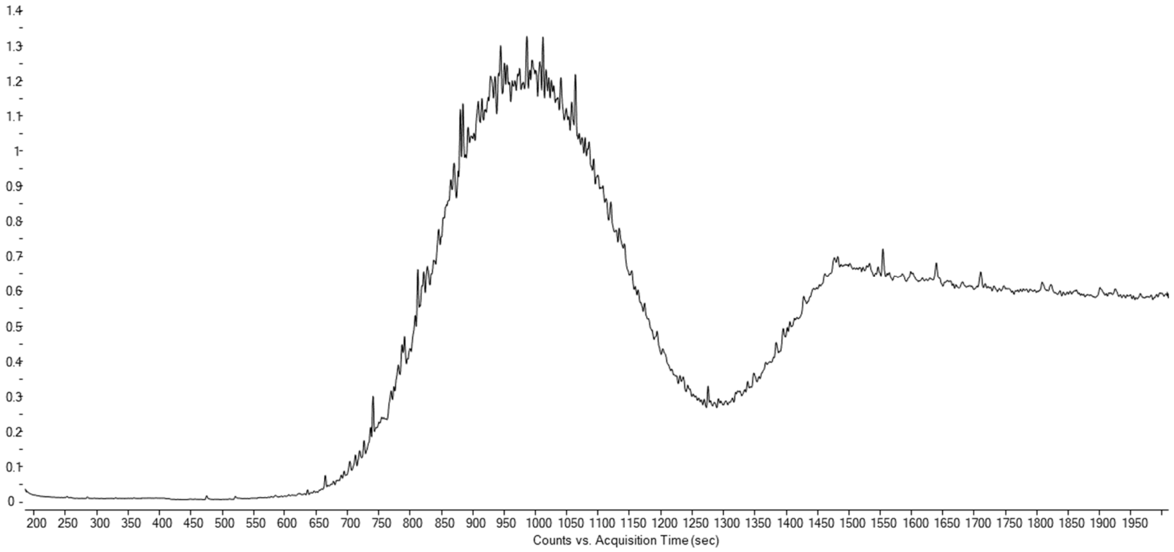

Figure S12. Chromatogram of acetone oily extract by GC/MS technique for Inconel 718. First metal ultrasonic treatment.

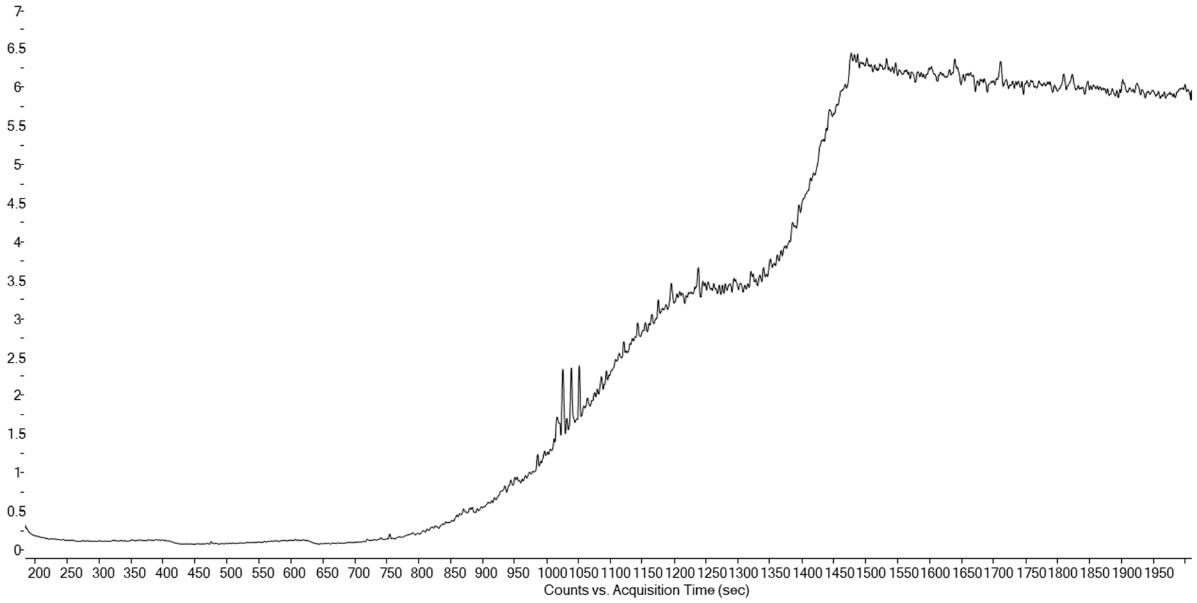

Figure S12. Chromatogram of acetone oily extract by GC/MS technique for Inconel 718. First metal ultrasonic treatment.

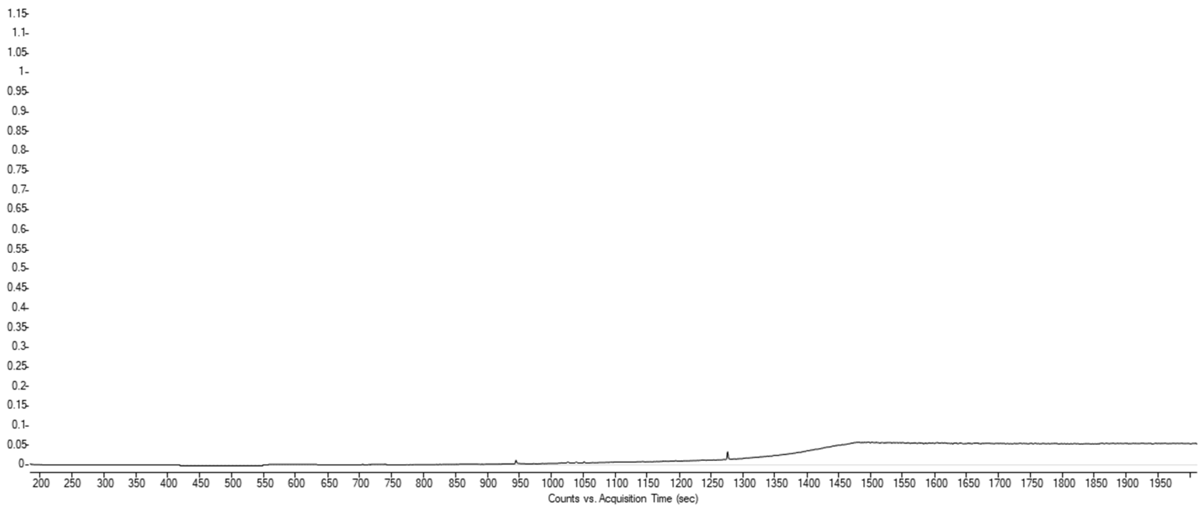

Figure S13. Chromatogram of acetone oily extract by GC/MS technique for Inconel 625, 718 and Titanium Grade 5. Second metal ultrasonic treatment.

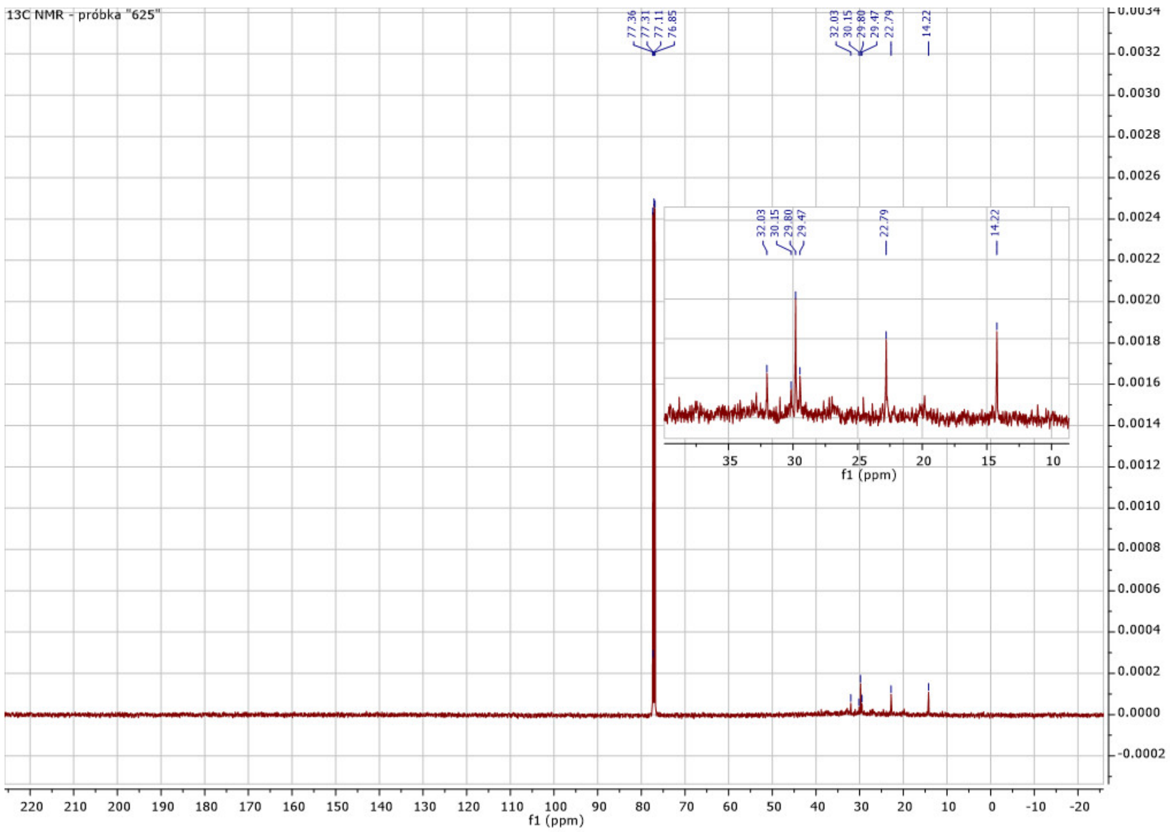

Figure S14. The 13C NMR spectrum obtained by extraction of the organic fraction of the Inconel 625 alloy.

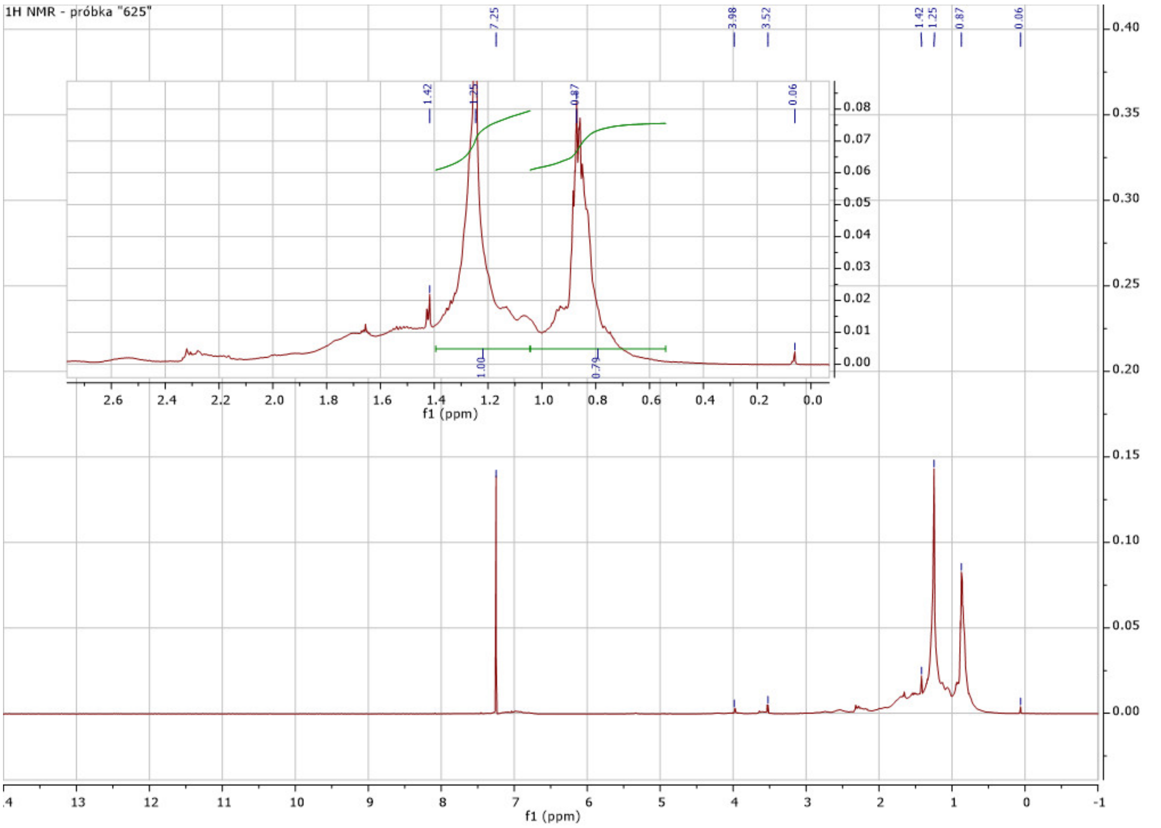

Figure S15. The 1H NMR spectrum obtained by extraction of the organic fraction of the Inconel 625 alloy.

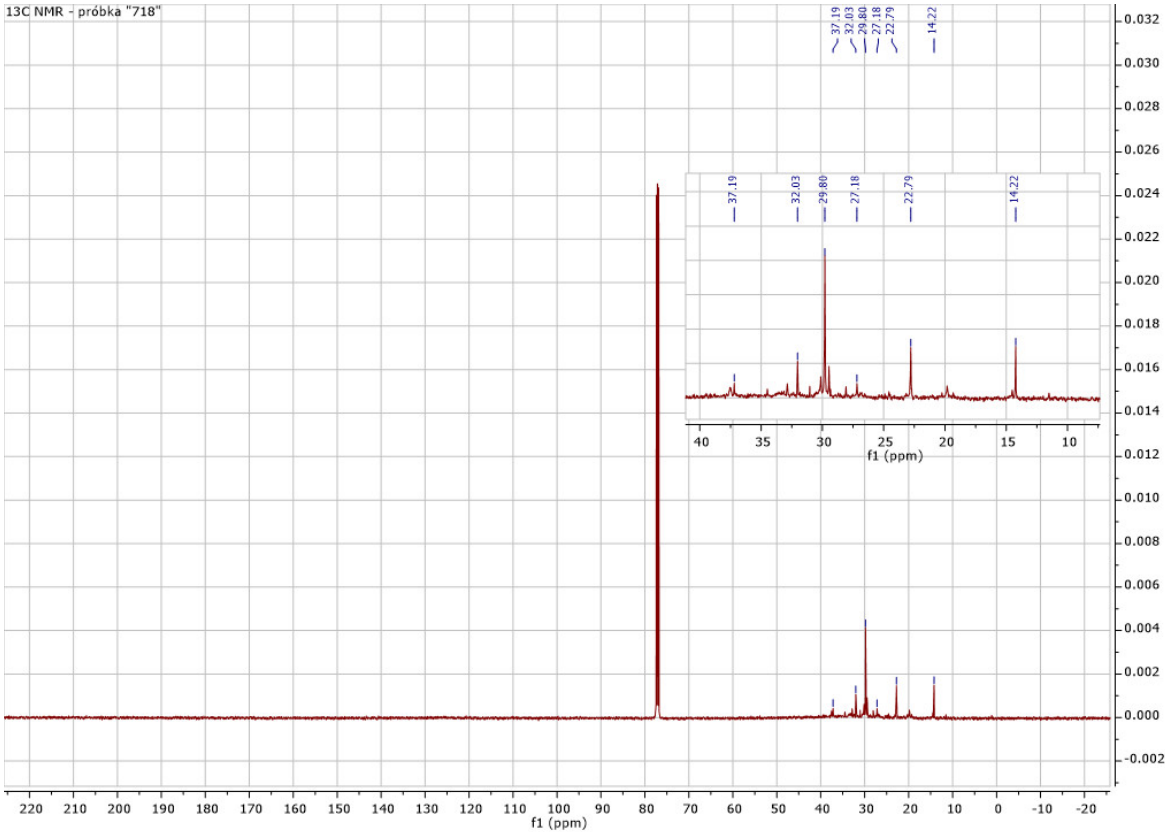

Figure S16. The 13C NMR spectrum obtained by extraction of the organic fraction of the Inconel 718 alloy.

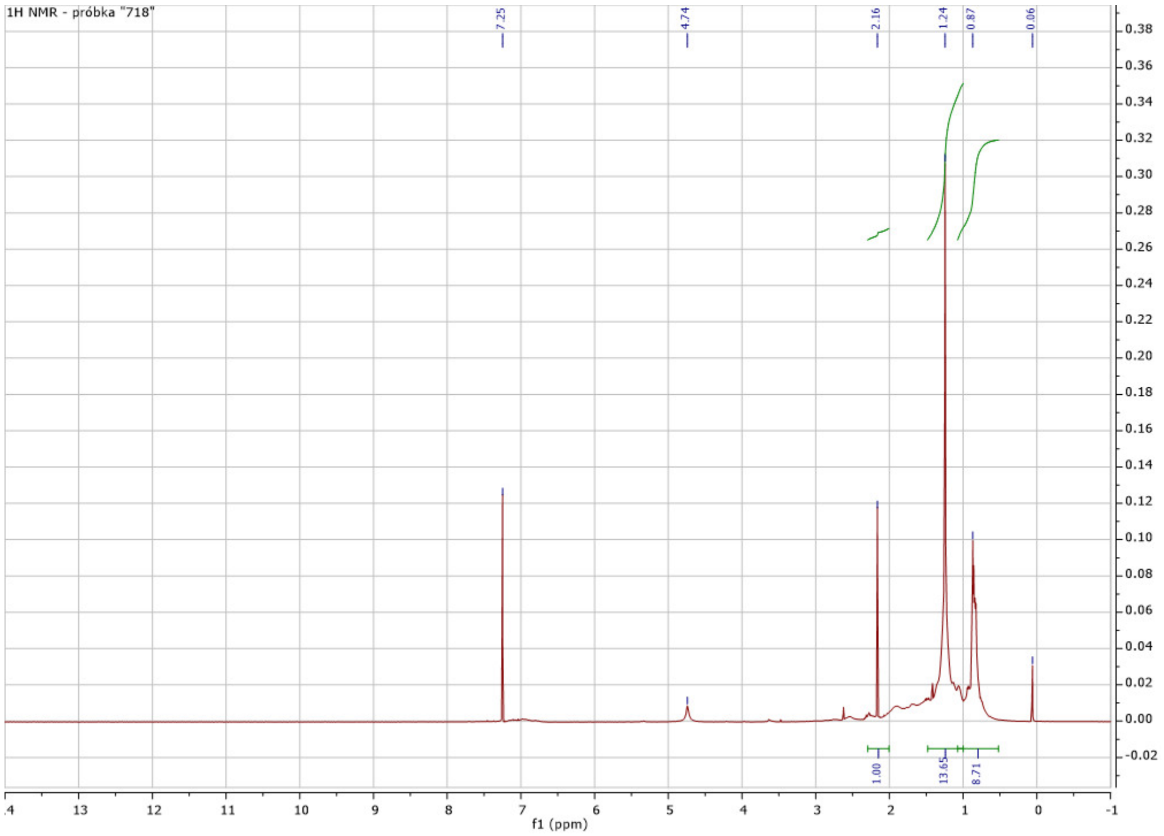

Figure S17. The 1H NMR spectrum obtained by extraction of the organic fraction of the Inconel 718 alloy.

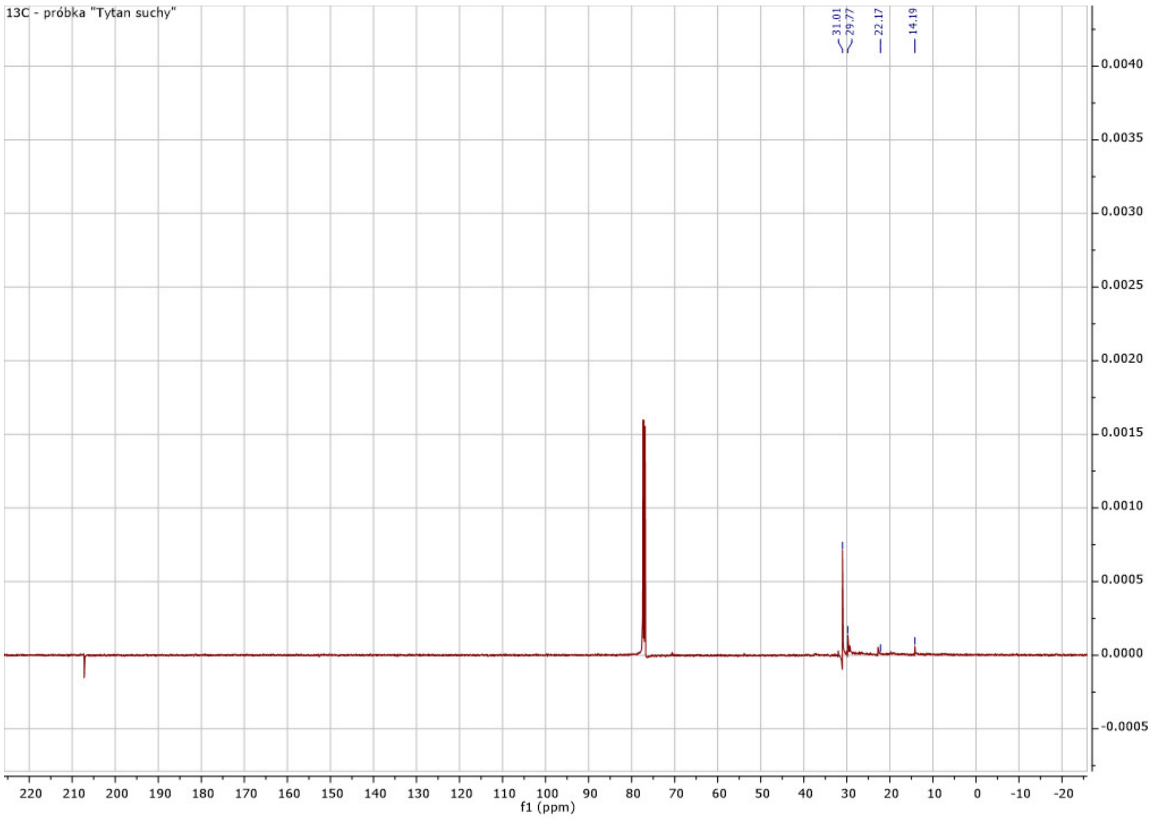

Figure S18. The 13C NMR spectrum obtained by extraction of the organic fraction of the Titanium Grade 5 alloy.

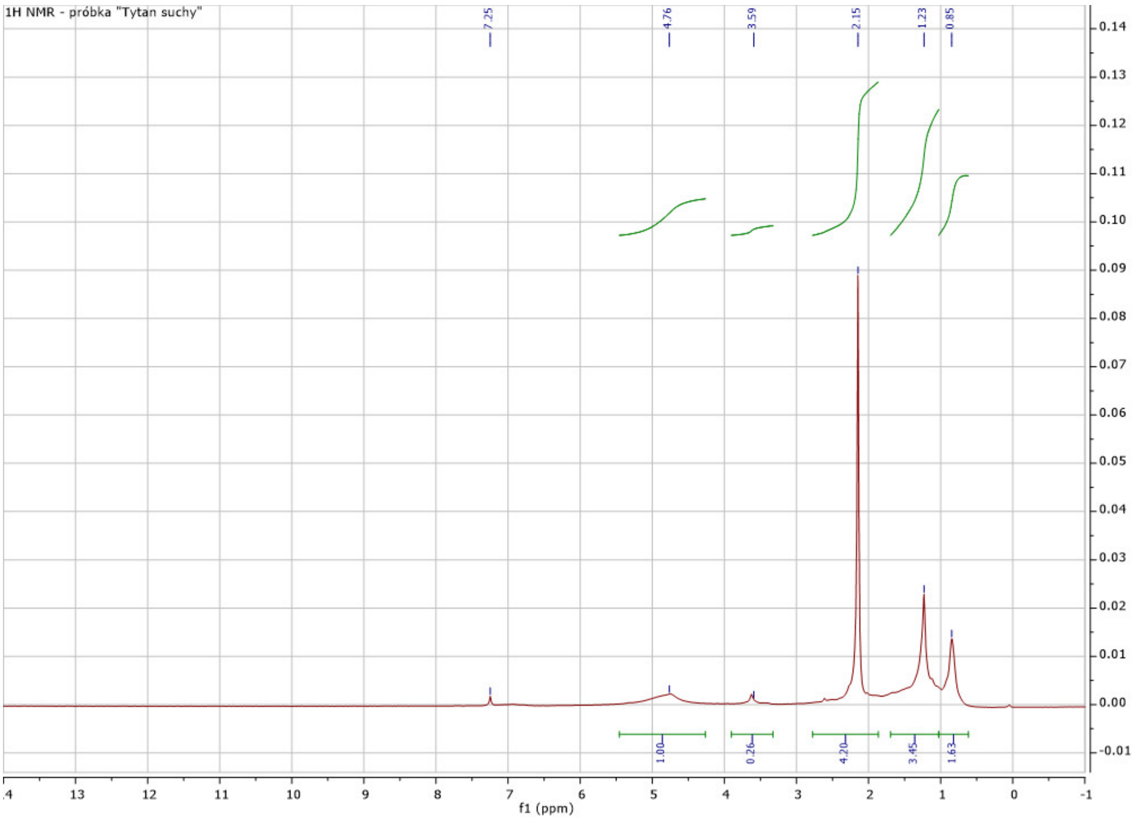

Figure S19. The 1H NMR spectrum obtained by extraction of the organic fraction of the Titanium Grade 5 alloy.

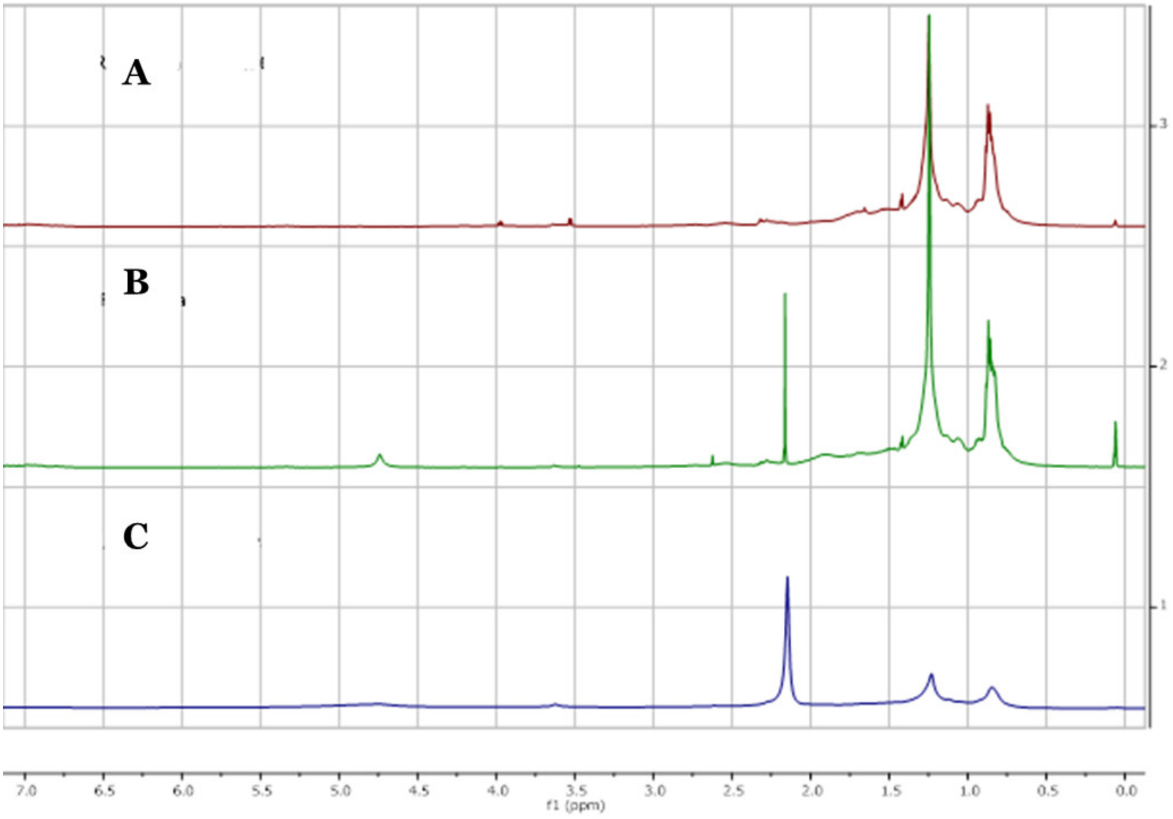

Figure S20. The comparison of 1H NMR spectra of the analysed organic fractions: (**A**) Inconel 625; (**B**) Inconel 718; (**C**) Titanium Grade 5, indicates their high chemical similarity.

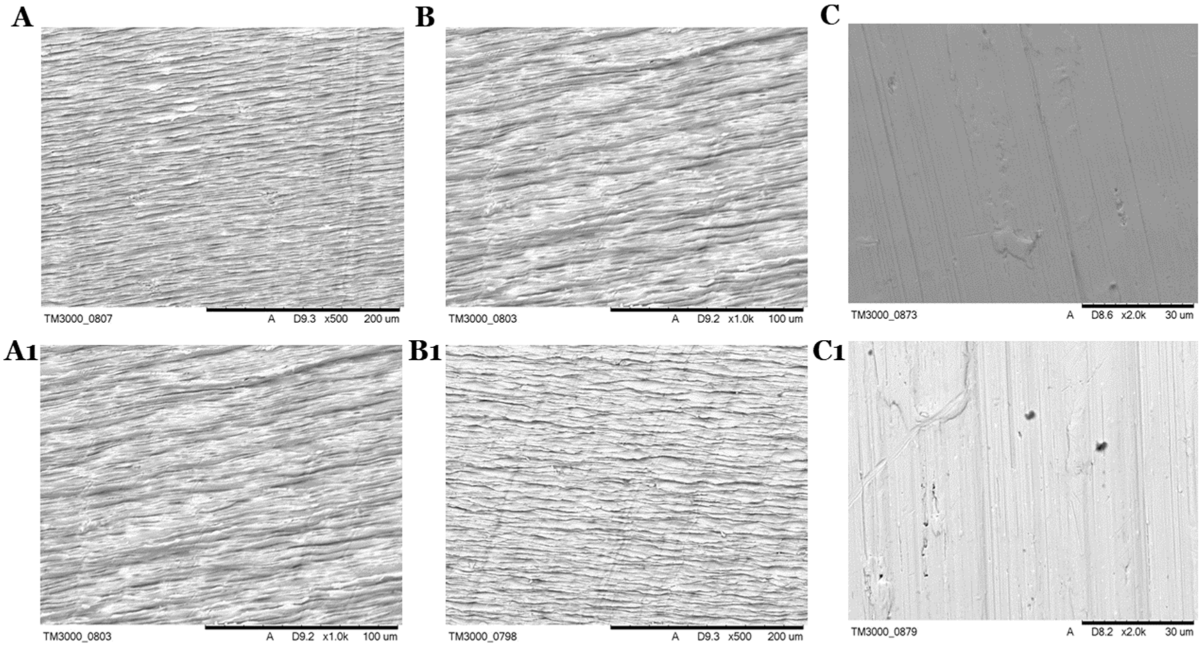

Figure S21. SEM-EDS photos after oil removal from Inconel 625 swarfs by washing with Spirdane D25 after 20 (**A**-dark side; **A1**-bright side), 40 (**B**-dark side; **B1**-bright side), and 60 (**C**-dark side; **C1**-bright side) minutes.

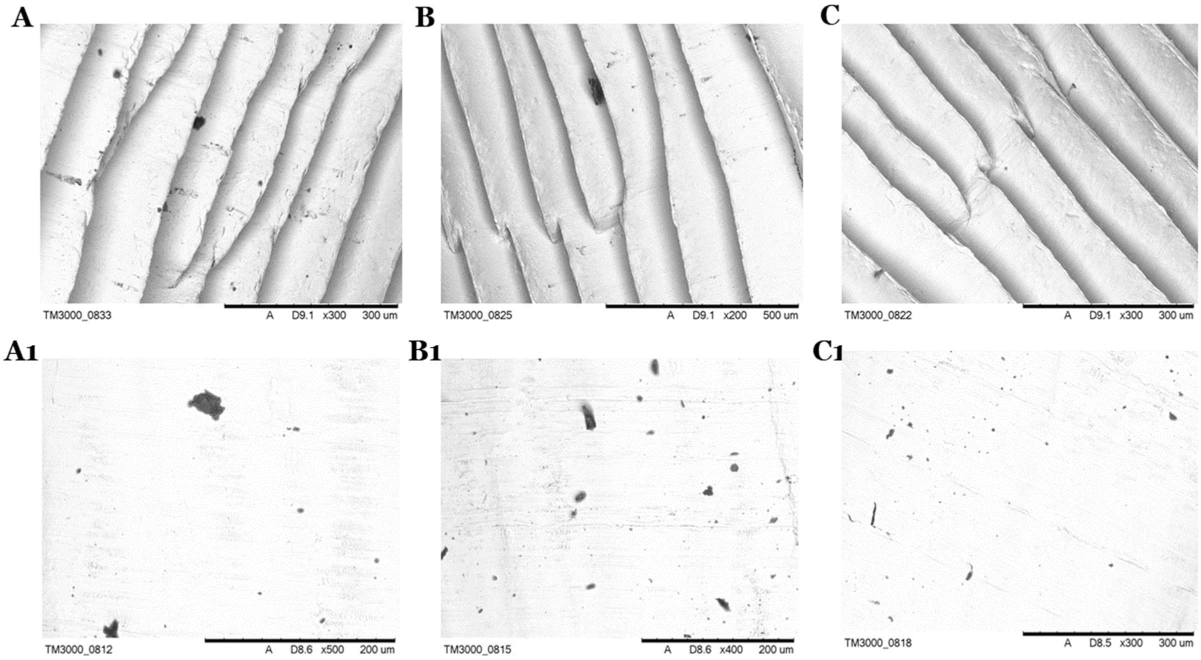

Figure S22. SEM-EDS photos after oil removal from Inconel 718 swarfs by washing with Spirdane D25 after 20 (**A**-dark side; **A1**-bright side), 40 (**B**-dark side; **B1**-bright side), and 60 (**C**-dark side; **C1**-bright side) minutes.

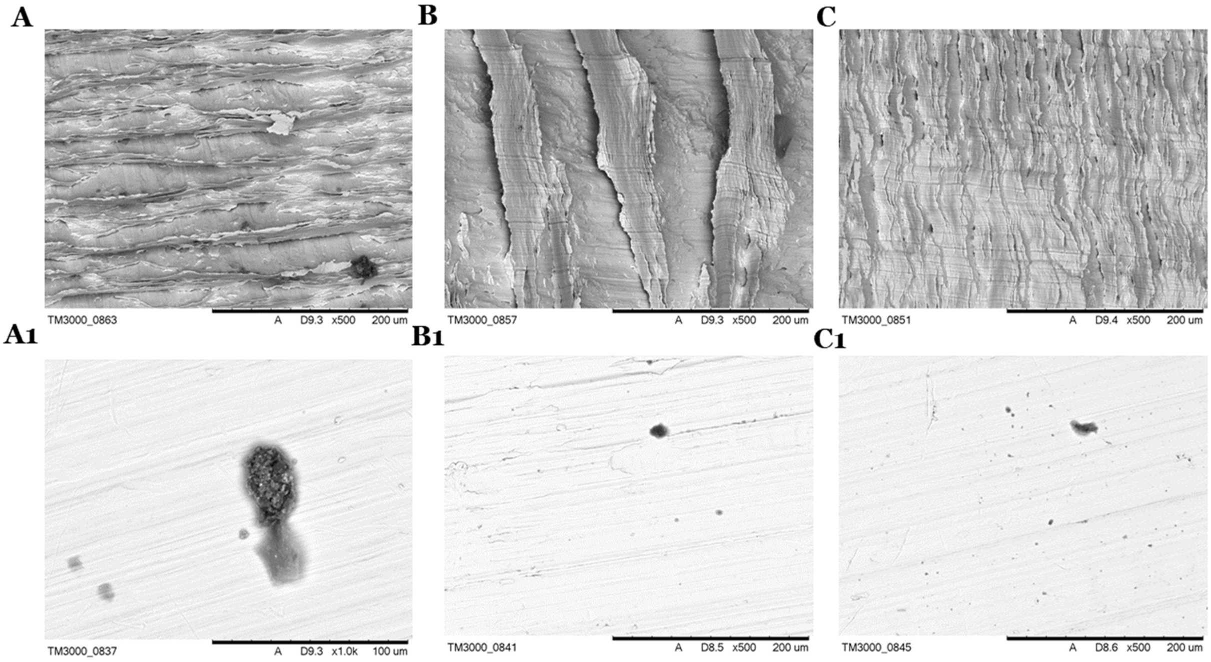

Figure S23. SEM-EDS photos after oil removal from Titanium Grade 5 swarfs by washing with Spirdane D25 after 20 (**A**-dark side; **A1**-bright side), 40 (**B**-dark side; **B1**-bright side), and 60 (**C**-dark side; **C1**-bright side) minutes.

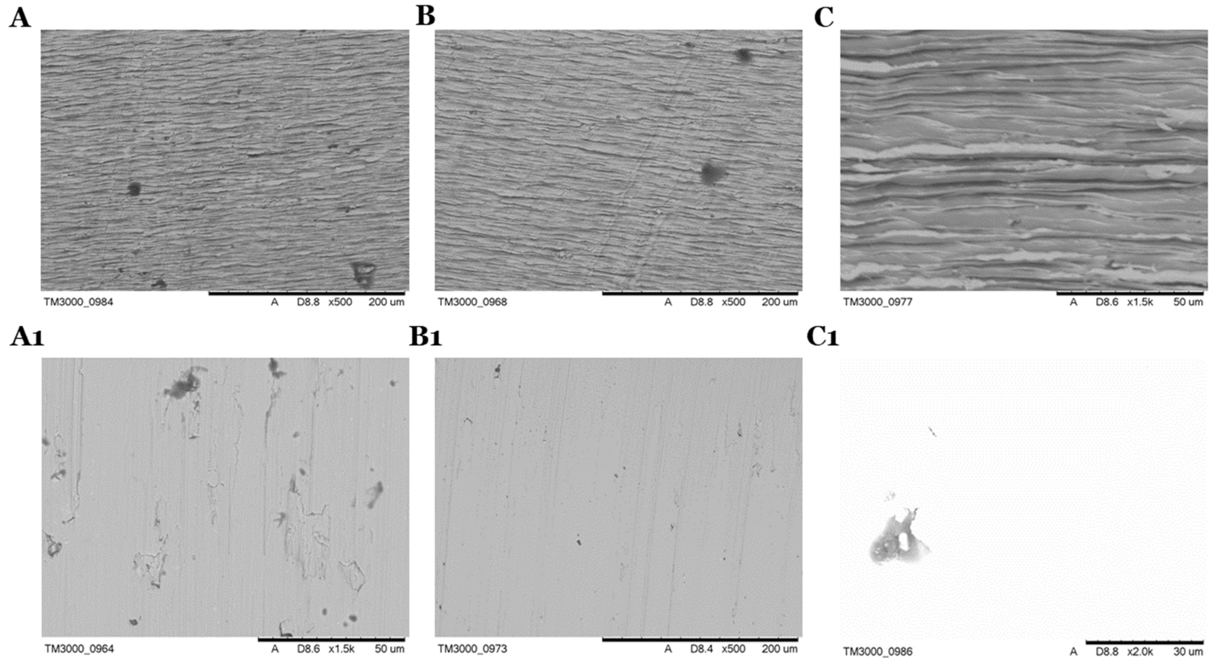

Figure S24. SEM-EDS photos after oil removal from Inconel 625 swarfs by washing with Spirdane D40 after 20 (**A**-dark side; **A1**-bright side), 40 (**B**-dark side; **B1**-bright side), and 60 (**C**-dark side; **C1**-bright side) minutes.

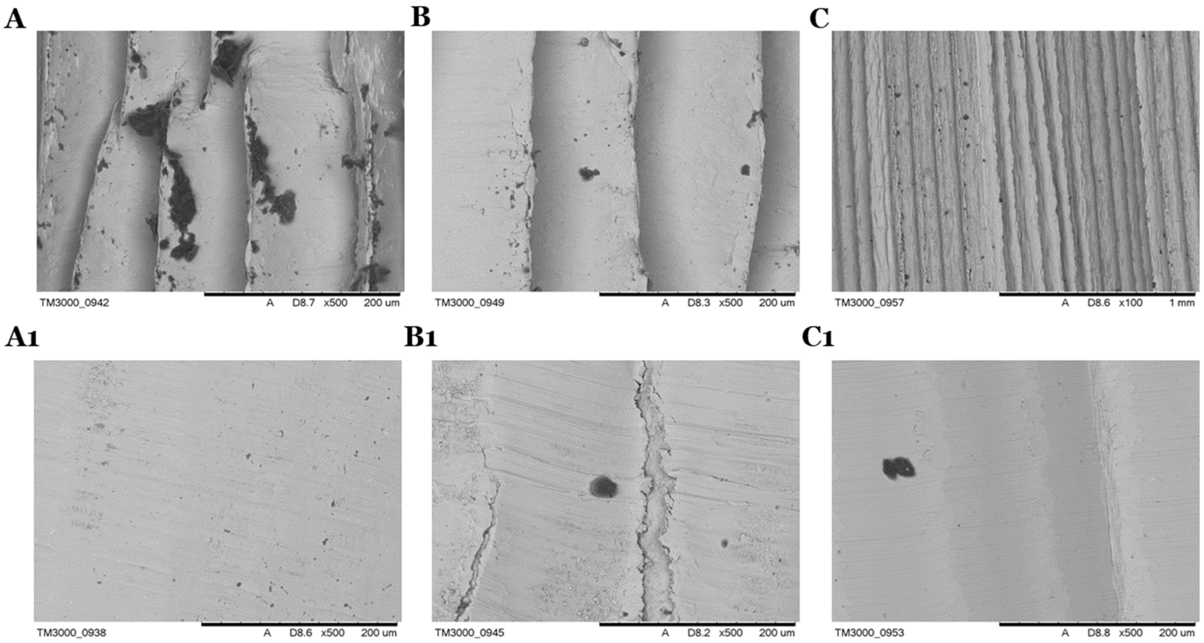

Figure S25. SEM-EDS photos after oil removal from Inconel 718 swarfs by washing with Spirdane D40 after 20 (**A**-dark side; **A1**-bright side), 40 (**B**-dark side; **B1**-bright side), and 60 (**C**-dark side; **C1**-bright side) minutes.

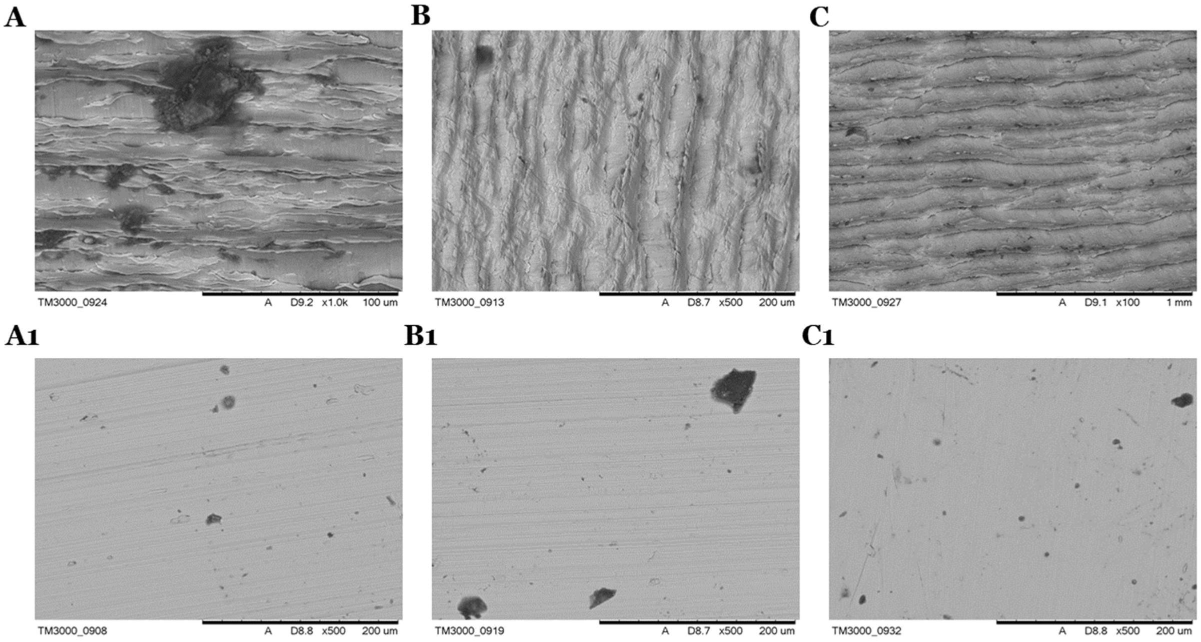

Figure S26. SEM-EDS photos after oil removal from Titanium Grade 5 swarfs by washing with Spirdane D40 after 20 (**A**-dark side; **A1**-bright side), 40 (**B**-dark side; **B1**-bright side), and 60 (**C**-dark side; **C1**-bright side) minutes.

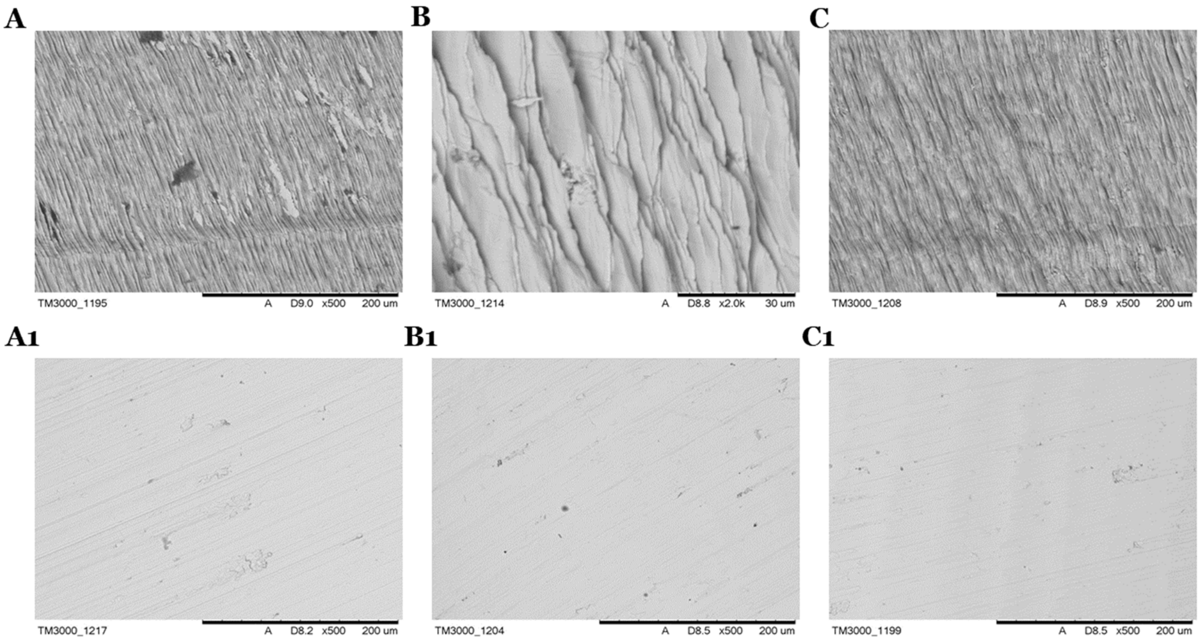

Figure S27. SEM-EDS photos after oil removal from Inconel 625 swarfs by washing with Spirdane D60 after 20 (**A**-dark side; **A1**-bright side), 40 (**B**-dark side; **B1**-bright side), and 60 (**C**-dark side; **C1**-bright side) minutes.

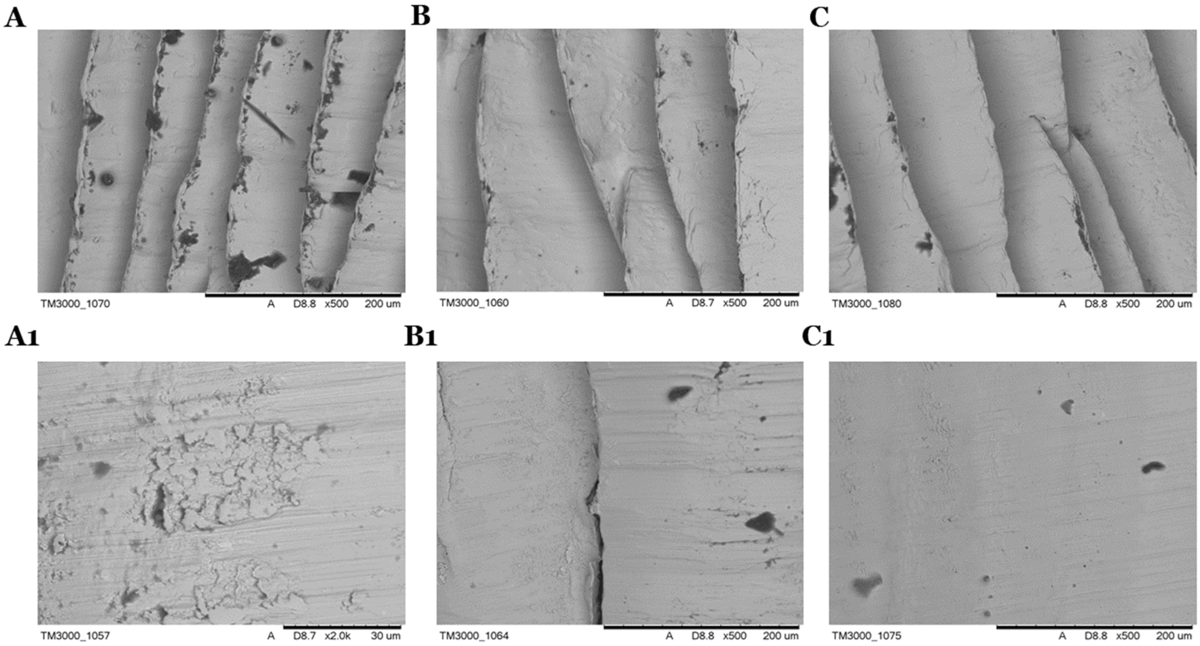

Figure S28. SEM-EDS photos after oil removal from Inconel 718 swarfs by washing with Spirdane D60 after 20 (**A**-dark side; **A1**-bright side), 40 (**B**-dark side; **B1**-bright side), and 60 (**C**-dark side; **C1**-bright side) minutes.

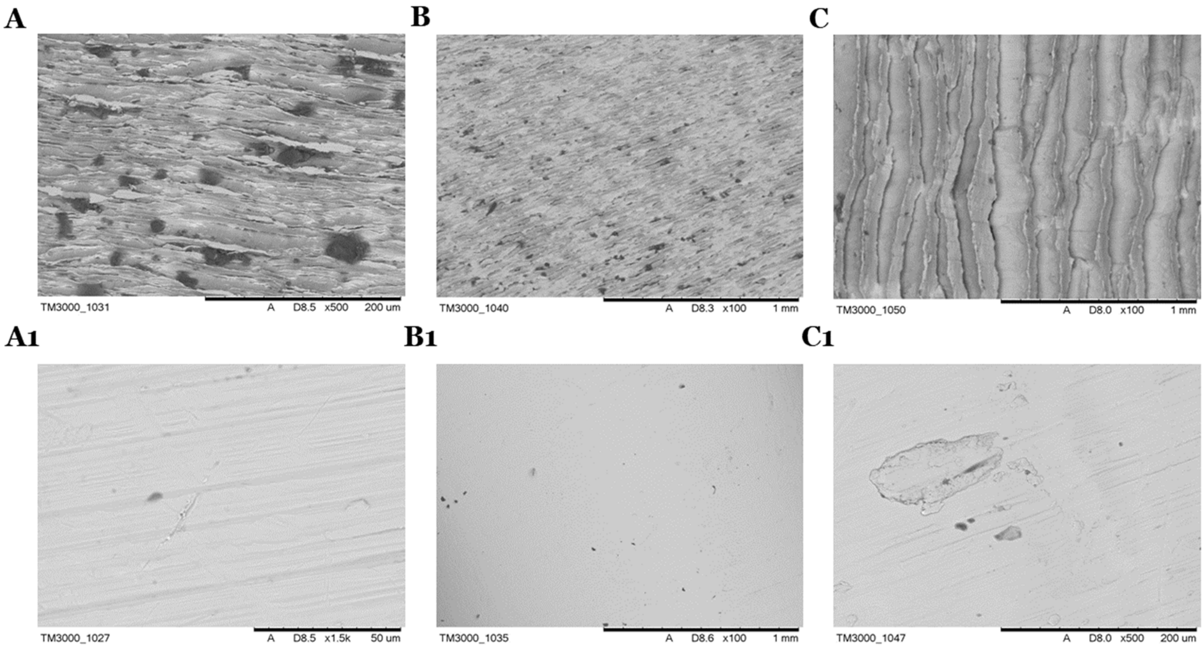

Figure S29. SEM-EDS photos after oil removal from Titanium Grade 5 swarfs by washing with Spirdane D60 after 20 (**A**-dark side; **A1**-bright side), 40 (**B**-dark side; **B1**-bright side), and 60 (**C**-dark side; **C1**-bright side) minutes.
